# Secretion and Function of Pituitary Prolactin in Evolutionary Perspective

**DOI:** 10.3389/fnins.2020.00621

**Published:** 2020-06-16

**Authors:** Arpád Dobolyi, Szilvia Oláh, Dávid Keller, Rashmi Kumari, Emese A. Fazekas, Vivien Csikós, Éva Renner, Melinda Cservenák

**Affiliations:** ^1^MTA-ELTE Laboratory of Molecular and Systems Neurobiology, Department of Physiology and Neurobiology, Institute of Biology, Eötvös Loránd University, Budapest, Hungary; ^2^Laboratory of Neuromorphology, Department of Anatomy, Histology and Embryology, Semmelweis University, Budapest, Hungary; ^3^Human Brain Tissue Bank and Microdissection Laboratory, Semmelweis University, Budapest, Hungary

**Keywords:** evolution, comparative, neuroendocrinology, hypothalamus, dopamine, osmoregulation, lactation

## Abstract

The hypothalamo-pituitary system developed in early vertebrates. Prolactin is an ancient vertebrate hormone released from the pituitary that exerts particularly diverse functions. The purpose of the review is to take a comparative approach in the description of prolactin, its secretion from pituitary lactotrophs, and hormonal functions. Since the reproductive and osmoregulatory roles of prolactin are best established in a variety of species, these functions are the primary subjects of discussion. Different types of prolactin and prolactin receptors developed during vertebrate evolution, which will be described in this review. The signal transduction of prolactin receptors is well conserved among vertebrates enabling us to describe the whole subphylum. Then, the review focuses on the regulation of prolactin release in mammals as we have the most knowledge on this class of vertebrates. Prolactin secretion in response to different reproductive stimuli, such as estrogen-induced release, mating, pregnancy and suckling is detailed. Reproduction in birds is different from that in mammals in several aspects. Prolactin is released during incubation in avian species whose regulation and functional significance are discussed. Little information is available on prolactin in reptiles and amphibians; therefore, they are mentioned only in specific cases to explain certain evolutionary aspects. In turn, the osmoregulatory function of prolactin is well established in fish. The different types of pituitary prolactin in fish play particularly important roles in the adaptation of eutherian species to fresh water environments. To achieve this function, prolactin is released from lactotrophs in hyposmolarity, as they are directly osmosensitive in fish. In turn, the released prolactin acts on branchial epithelia, especially ionocytes of the gill to retain salt and excrete water. This review will highlight the points where comparative data give new ideas or suggest new approaches for investigation in other taxa.

## Introduction

Prolactin is an ancient regulatory molecule with diverse regulatory functions (Freeman et al., 2000). Prolactin has been shown to be expressed in a variety of different organs, however, its expression level is highest in the pituitary (Bu et al., 2015). It was suggested that in early vertebrates, the expression of prolactin was more diverse, but even in fish, it is already predominantly expressed in specific cells of the adenohypophysis, from which prolactin is released into the bloodstream to act as a multifunctional hormone. Based on the structure and receptor type of prolactin, it belongs to the cytokines. Thus, together with growth hormone, it forms a group of pituitary hormones, which are not a 3–51 amino acid long neuropeptides acting on G-protein coupled receptors, but possess 1 transmembrane domain cytokine receptor. In these properties, prolactin and growth hormone are also different from the structure of other peptide and glycoprotein pituitary hormones. Another unique property of prolactin among adenohypophyseal hormones is that it does not have a target endocrine gland, which would mediate its actions but rather it exerts its actions directly via prolactin receptors localized in a variety of different target organs (Grattan, 2015). Most of the major targets of prolactin are epithelial cells, on which prolactin can exert proliferative effects as well as faster gene expression and even faster molecular actions (Aoki et al., 2019). In this paper, we will review the major actions of prolactin in vertebrate taxa. Most knowledge is available in mammals and fish where lactation and osmoregulation are the most established functions of prolactin, respectively (Horseman and Gregerson, 2014). Data are also accumulating in birds where prolactin is critically important in parental behavioral control (Smiley, 2019). The different functions require diverse stimuli for the release of prolactin. Our knowledge is more limited in this aspect of prolactin regulation. An aim of this review is to compare prolactin-related regulations between different vertebrate taxa to generate new research approaches.

## Prolactin and the Evolution of Prolactin Genes

**FIGURE 1:**
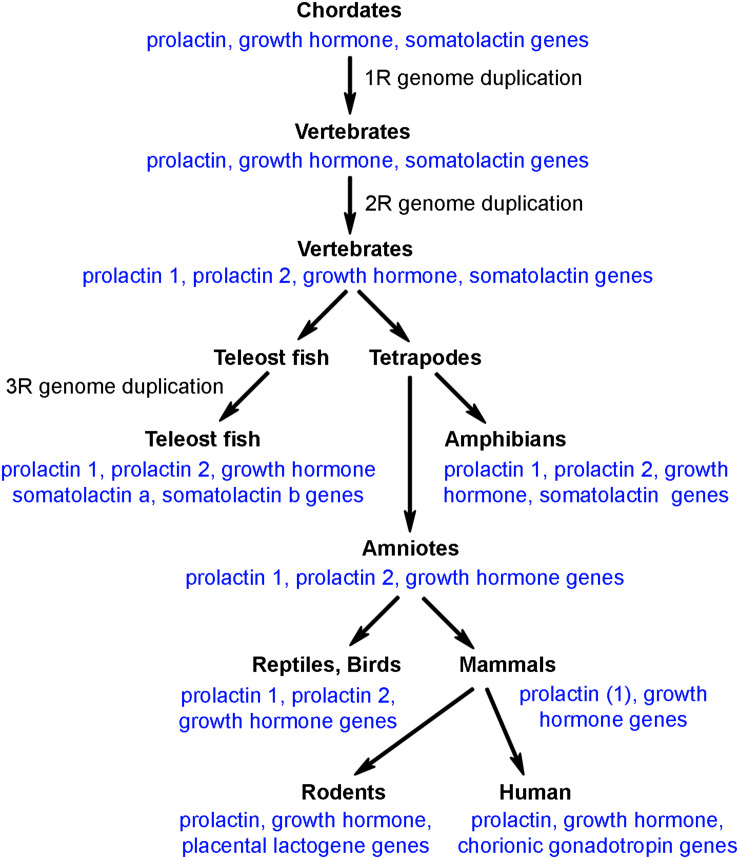
The presence of prolactin/growth hormone family of genes in different clades of vertebrates.

Prolactin belongs to a gene family that comprises prolactin, growth hormone and somatolactin. The peptide sequences of these proteins exhibit approximately 20% homologies to each other in teleost species where all 3 of them are present. There are several additional versions of prolactin in specific vertebrate species, which were formed by local gene duplication. Although some reports suggested the presence of prolactin-like peptides in invertebrates, the general agreement is that the whole protein family was formed in chordates. It is known that whole genome duplication occurred 3 times during vertebrate evolution; the first round (1R) at the transition from chordates to vertebrates, the second round (2R) at the transition from agnathans to gnathostomes, and the third round (3R) after divergence of the teleost lineage. As for the prolactin gene family, however, even ancient chordate species before the 1R whole genome duplication possessed the three genes (Figure 1). Following 1R and 2R whole genome duplications, only one additional gene, prolactin 2 emerged while the teleost specific 3R whole genome duplication resulted in another gene, somatolactin b. In turn, somatolactin was lost in amniotes, and prolactin 2 was lost in mammals (Ocampo Daza and Larhammar, 2018a). The two different prolactins in teleost fish have similar functions. The prolactins are co-secreted from lactotrophs (Specker et al., 1993) and may bind to the same prolactin receptor albeit with different affinities (Auperin et al., 1995). The change in prolactin sequence showed an uneven speed in vertebrate evolution, which is in line with its role in major adaptive events, such as freshwater adaptation, or the development of lactation in mammals. Furthermore, new prolactin-like protein coding genes have developed, which, however, are typically not expressed in the pituitary. For example, rodents possess several placental lactogens (Bridges et al., 1996; Goffin et al., 1996). However, the present review, focuses on pituitary prolactin and mentions other prolactin-related proteins only if necessary for understanding of the functions of pituitary-derived prolactin.

The pituitary itself is an organ unique to vertebrates. In cephalochordates, the pituitary has a homolog, Hatschek’s pit, an organ in the epithelial invagination of the oral cavity whose morphology and development is similar to the Rathke’s pouch of the vertebrate embryo (Kubokawa et al., 2010). Cells of the Hatschek’s pit express some proteins homologous to some adenohypophyseal hormones but not to prolactin (Holland et al., 2008). In vertebrates, the structure of the pituitary is similar in all classes (Sage and Bern, 1971). This structure consists of an anterior lobe, an intermediate lobe and a posterior lobe, or neurohypophysis. The anterior lobe of the pituitary is the adenohypophysis, which contains lactotrophs as well as other types of secretory cells (Bern and Nicoll, 1968). Lactotrophs actually form a more definitive mass within the rostral pars distalis of the adenohypophysis as opposed to the more intermingled localization of lactotrophs with other hormone producing cells in the lungfish and tetrapod pituitary (Manzon, 2002). The secretory activity of adenohypophyseal cells is regulated by releasing and inhibitory factors of hypothalamic origin. Tetrapods have a hypophyseal portal system, which is reached by hypothalamic hormones in the median eminence. Fish do not have a median eminence, and the anterior part of the neurohypophysis was suggested to play a similar role in them (Sage and Bern, 1971).

## Lactotroph Cells in the Pituitary and Their Prolactin Release

Lactotrophs are located in the anterior lobe of the pituitary. In fish, prolactin secretion from lactotrophs seems to be affected directly by osmolarity as even cultured lactotrophs are sensitive to reduced osmolarity in their extracellular environment and respond to it by increased size and prolactin secretion (Labella et al., 1975; Weber et al., 2004). In mammals, in contrast, lactotrophs spontaneously release prolactin. It has been demonstrated that lactotrophs fired spontaneous plateau-bursting action potentials, which generated high amplitude calcium signals due to calcium influx via voltage-gated calcium channels (Van Goor et al., 2001). While different hypothalamic prolactin releasing factors have been proposed in mammals – the most compelling evidence is available for thyrotropin-releasing hormone -, it is still a general consensus that prolactin secretion from lactotrophs is only controlled by the inhibitory action of dopamine exerted by D2 dopamine receptors in mammals. The G-protein coupled D2 receptors utilize different signal transduction pathways to inhibit spontaneous prolactin release. They block voltage-gated calcium channels via pertussis toxin sensitive G_*i/o*_ proteins while also desensitize calcium ion secretion coupling via pertussis toxin insensitive Gz proteins (Gonzalez-Iglesias et al., 2008). Accordingly, D2 receptor agonists, e.g., bromocriptine, block spontaneous electrical activity of lactotrophs as well as accompanied prolactin release (Auriemma et al., 2019). Therefore, bromocriptine can be used to cease milk production or treat hyperprolactinemia induced by pituitary tumors. In addition, mice lacking the D2 receptor are hyperprolactinemic (Saiardi et al., 1997). Dopamine reaches lactotrophs from the portal circulation of the pituitary, which contains dopamine following its release from dopaminergic neurons located in the arcuate nucleus (A12 dopaminergic cell group in mammals). Additional alternative ways of dopamine reaching lactotrophs have also been suggested, notably dopaminergic neurons located in the periventricular area (A14 dopaminergic cell group in mammals) projecting to the intermediate lobe of the pituitary. Although D2 receptors on lactotrophs play a role in the control of prolactin secretion in birds (Lv et al., 2018), they seem to possess a prolactin-releasing hormone, vasoactive intestinal peptide (VIP) (Tong et al., 1998; Christensen and Vleck, 2008)- Contrary to the lack of active prolactin-releasing mechanisms in mammals. VIP, similar to dopamine, is also released from neurons located in the infundibular/arcuate nucleus (Kosonsiriluk et al., 2008). The mechanism of negative feedback regulation for stable prolactin levels is driven by prolactin itself, which increases the neuronal activity of neuroendocrine dopaminergic neurons (Brown et al., 2012) and also their dopamine synthesis (Arbogast and Voogt, 1995) to reduce prolactin secretion. In birds, the participation of VIP in negative feedback would also make sense as an additional mechanism of feedback inhibition of prolactin release, but the available evidence is scarce (Namken et al., 2017). In mammals, VIP has also been suggested as regulator of prolactin secretion. VIP was found to be synthesized both in the hypothalamus and pituitary (Lam, 1991). *In vitro*, VIP stimulated prolactin release from lactotrophs (Bjoro et al., 1990). However, its effect was not specific as it also released other pituitary hormones (Lam, 1991). More recently it was suggested that VIP of suprachiasmatic origin could affect circadian rhythm of prolactin secretion (Egli et al., 2004; Kennett et al., 2008), or only some subtypes of lactotrophs could be affected by VIP (Christian et al., 2007), alternatively, VIP could affect proliferation of lactotrophs (Carretero et al., 2006). But most likely, VIP is not a physiological regulator of prolactin secretion in mammals (Phillipps et al., 2019). It is also not established yet if prolactin release in fish is regulated by the hypothalamus. It seems likely that not only osmolarity in the pituitary but other factors play a part in prolactin secretion. Estrogen evoked prolactin release from the marine teleost, sea bream (*Sparus aurata L.*), and this effect was inhibited by VIP (Brinca et al., 2003). Thus, VIP could be involved in the regulation of prolactin release as it is in avian species but exerts the opposite action by inhibiting the release.

## Prolactin Receptors and Their Signal Transduction

Prolactin is secreted from the pituitary to the circulation. Prolactin may bind to prolactin-binding proteins, which have been suggested in mammals but not in other taxa (Kline and Clevenger, 2001). Prolactin then exerts its actions by binding to its plasma membrane receptors.

### Evolution of Prolactin Receptors

DNA sequence comparisons as well as synthetic analysis revealed that early vertebrates possessed a common growth hormone/prolactin receptor even after the 2R tetraploidization event (even though separate growth hormone and prolactin genes were present before 1R). The separate prolactin receptor appeared soon after that by gene duplication (Ocampo Daza and Larhammar, 2018b). The teleost-specific 3R tetraploidization resulted in 2 prolactin receptor genes (PrlRa and b), which is characteristic of most teleost fish.

### Signal Transduction of Prolactin

The prolactin receptors all belong to the type I cytokine receptor family (Bole-Feysot et al., 1998). In mammals, there are long, intermediate and short prolactin receptor isoforms generated by alternative splicing (Freeman et al., 2000). All these isoforms are 1 transmembrane domain plasma membrane receptors. Signal transduction requires dimerization of the receptors. The receptors do not have enzyme activity but rather attract adaptor molecules upon prolactin binding to initiate signal transduction. There are two major types of signal transduction pathways for the prolactin receptor. The conventional pathway uses a cytosolic tyrosine kinase, Janus kinase 2 (Campbell et al., 1994), which phosphorylates signal transducer and activator (STAT) 5 (Clevenger and Kline, 2001). Phosphorylated STAT5 (pSTAT5) then forms homodimers and acts as a transcription factor to induce the expression of various proteins, e.g., casein milk proteins. An alternative pathway is the mitogen-activated protein (MAP) kinase pathway, which generally mediates the proliferative actions of prolactin (Radhakrishnan et al., 2012).

### Expression of Prolactin Receptors in Different Tissues

Prolactin receptors are expressed in a variety of different organs based on different blotting and PCR technologies. In the fish, the osmoregulatory organs, such as the gills, kidney, and intestine contained the highest amount of prolactin receptor (Lee et al., 2006). In addition, prolactin receptors are also abundant in the brain, liver, gonads, liver, spleen, and present in the heart, muscle, bone, and skin, too (Sandra et al., 2000; Santos et al., 2001). Prolactin receptor has a similarly widespread tissue distribution in other clades, too. In mammals, the long and the short forms of prolactin receptors had similar distribution patterns with the long form dominating in most organs except for the kidney and lung (Ouhtit et al., 1993). More detailed distributional patterns of prolactin receptors within the organs expressing it have also been established, which, for example, indicated particularly high level of prolactin receptors within ionocytes of gill epithelium in fish, or alveolar cells of the mammary gland.

### Distribution of Prolactin Receptors in the Brain

**FIGURE 2:**
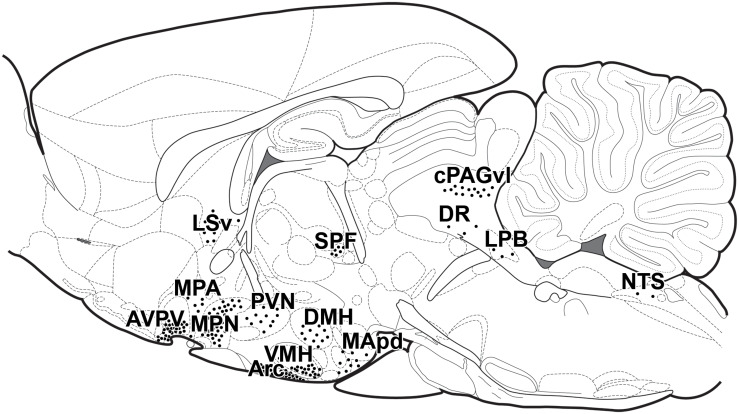
Distribution of prolactin sensitive neurons in lactating mothers in schematic sagittal section. Each dot represents 20 pSTAT5-positive neurons evoked by suckling in lactating mice, with the exception of Arc, where each dot represents 85 neurons/mm^2^ in a 40 mm thick section (Olah et al., 2018). This distribution of pSTAT5-positive cells is essentially the same as the distribution of exogenous prolactin-evoked pSTAT5 and of prolactin receptors visualized by *in situ* hybridization histochemistry (Brown et al., 2010). Arc, arcuate nucleus; AVPV, anteroventral periventricular nucleus; DMH, dorsomedial hypothalamic nucleus; DR, dorsal raphe nucleus; cPAGvl, caudal periaqueductal gray, ventrolateral subdivision; LPB, lateral parabrachial nucleus; LSv, lateral septal nucleus, ventral subdivision; MApd, medial amygdaloid nucleus, posterodorsal subdivision; MPA, medial preoptic area; MPN, medial preoptic nucleus; NTS, nucleus of the solitary tract; PVN, paraventricular hypothalamic nucleus; SPF, subparafascicular area; VMHvl, ventromedial hypothalamic nucleus, ventrolateral subdivision.

Given the enormously high number of different cell types in the brain and the different functions connected to the nervous system, the distribution of prolactin receptors within the mammalian brain has been in focus for decades. Initial immunolabeling studies demonstrated neuronal expression and a topographical distribution within the brain with high level of prolactin receptor in the anteroventral periventricular nucleus, the medial preoptic area, the paraventricular, and the arcuate nuclei with even more hypothalamic sites becoming visible in lactating rats (Pi and Voogt, 2000) with weak labeling in some striatal, thalamic and cortical sites, too. This distributional pattern was confirmed by *in situ* hybridization histochemistry, which also showed that the short form of the receptor may be present in hypothalamic but also in extrahypothalamic sites (Bakowska and Morrell, 1997, 2003). Modern molecular biological techniques using prolactin receptor-Cre recombinase mice bred with green fluorescent protein reporter mice revealed the precise expression of the long form of the prolactin receptor in the same sites as also described by *in situ* hybridization histochemistry (Kokay et al., 2018). The sites of signal transduction of prolactin can also be directly examined using pSTAT5 immunohistochemistry, which resulted in the same labeling pattern following injection of exogenous prolactin (Brown et al., 2010; Sjoeholm et al., 2011) or following suckling in lactating mice (Olah et al., 2018) as described with the above techniques (Figure 2). Some of these methods cannot properly address the subcellular location of prolactin receptors, e.g., where they are located in relation to synapses. Therefore, techniques, which can address these type of questions, such as immunohistochemistry remain important research tools in the field in the future, too.

## Reproductive Actions of Prolactin

The reproductive cycle of animals can be generally divided into sexual and parental phases. Despite the phylogenetic diversity in the specific regulations, it can be claimed that gonadotropins and sexual steroid hormones play pivotal roles in the control of the sexual phase while prolactin is the major regulator of the parental phase (Everett, 1964). Of course, the 2 phases are connected with each other. Indeed, prolactin is released during the luteinizing hormone surge in the estrous cycle, due to increased estrogen levels, which could be a direct pituitary action or a kisspeptin-evoked suppression of dopaminergic activity (Szawka et al., 2011; Grattan and Szawka, 2019). The role of the prolactin released during estrous is not well established to date (Phillipps et al., 2019) as it does not seem to affect female sexual behavior (Witcher and Freeman, 1985). Prolactin secretion is also induced by mating, both in males and females, and a characteristic prolactin secretory pattern appears during pregnancy. Our knowledge is highly limited in nonmammalian species regarding estrogen- or mating-induced prolactin release. Furthermore, dopamine release under these circumstances varies even within mammals, as discussed below.

### Prolactin Released During Mammalian Pregnancy

Mating induces prolactin secretion (Exton et al., 2001). Information on mating is probably conveyed to the hypothalamus in a neuronal pathway similar to that involved in suckling (described below) as both transfer somatosensory information from the spinal cord to the same hypothalamic site, the dopaminergic neurons in the arcuate nucleus. The role of mating-induced prolactin secretion is not known (Voogt et al., 2001). In males, it could contribute to the sexual refractory period or the formation of parental motivation. In females, mating-induced release may initiate the characteristic pattern of prolactin secretion during pregnancy. This secretory pattern can be highly different depending on the species. In humans, progesterone synthesis in the corpus luteum is maintained by chorionic gonadotropin of placental origin. In contrast, prolactin maintains the corpus luteum in rodents, which requires an immediate high concentration of prolactin in the serum (Phillipps et al., 2019). To this end, prolactin is released from the pituitary twice a day (Gunnet and Freeman, 1983). This secretory pattern is probably triggered by mating and lasts for 12–13 days if the animal is pseudopregnant, e.g., following artificial vaginocervical stimulation (Gunnet and Freeman, 1984). In turn, prolactin secretion from the pituitary ceases at approximately 9–10 days of pregnancy, as it is terminated by negative feedback due to lactogens of placental origin (Goffin et al., 1996). The placenta is fully functional by that time of pregnancy, and rodents have expanded the number of genes encoding prolactin-like proteins, which are expressed in the placenta (Soares et al., 2007). Some of these placental lactogens act on the prolactin receptor, therefore, their high serum concentration inhibits prolactin release from the pituitary by activating dopaminergic neurons in the arcuate nucleus via the prolactin receptor expressed in these neurons (Goffin et al., 1996). In contrast to rodents, human prolactin levels rise gradually during pregnancy, which is likely a steroid driven process (Phillipps et al., 2019). The major role of prolactin and placental lactogens in late pregnancy is mammopoesis. This function can be well assessed in mice lacking prolactin or prolactin receptor: mammary glands do not have proper side branching and alveologenesis, of which only the former can be rescued by progesterone (Horseman and Gregerson, 2014). These actions of prolactin are complex as they are mediated by receptor activator of nuclear factor kappa-B (RANK) present in secretory cells of the alveolar epithelium. RANK ligand is released from prolactin-sensing cells in the alveolar epithelium to mediate paracrine actions (Fernandez-Valdivia et al., 2009). The pregnancy of rats lasted for 22 days. Approximately 1 day before parturition, a prolactin surge emerges probably because progesterone is reduced at this point, which helps dopaminergic neurons escape feedback stimulation, even though placental lactogen levels are still high. It was proposed that the dopamine content of dopaminergic neurons in the arcuate nucleus is reduced (Andrews and Grattan, 2003), possibly producing encephalin instead of dopamine (Yip et al., 2019). The function of this release of prolactin before parturition is not clearly established because prolactin effects required during pregnancy, such as lactogenesis, adaptation of the brain for maternal behavior, or increased insulin secretion via prolactin receptors in the beta cells of the pancreas to avoid hyperglycemia can all be performed by placental lactogens.

### Prolactin Released in the Postpartum Period in Response to Suckling in Mammals

Serum prolactin levels are generally high in the postpartum period (Crowley, 2015). Although the proliferation and hypertrophy of lactotrophs are required for their maintenance (Le Tissier et al., 2015), some processes are required to prevent the negative feedback caused by prolactin itself. The mechanisms of these alterations during lactation have not been elucidated to date. The phenotypic changes described above for late pregnancy (that is an induced enkephalin production by tuberoinfundibular dopaminergic neurons) could be involved. In addition, insulin-like growth factor-1 (IGF-1) has also been suggested to play a role (Dobolyi and Leko, 2019). Prolonged intracerebroventricular IGF-1 was shown to stimulate dopamine secretion in the mediobasal hypothalamus and inhibit prolactin secretion from the pituitary (Leko et al., 2017a). In turn, a binding protein of IGF-1, IGF-binding protein-3 (IGFBP-3) is induced dramatically during lactation specifically in the arcuate nucleus (Leko et al., 2017b). Therefore, IGFBP-3 may be able to sequester IGF-1 from around dopaminergic neurons thereby eliminating the local stimulatory effects of otherwise elevated IGF-1 on dopaminergic neurons (Leko et al., 2017b).

**FIGURE 3:**
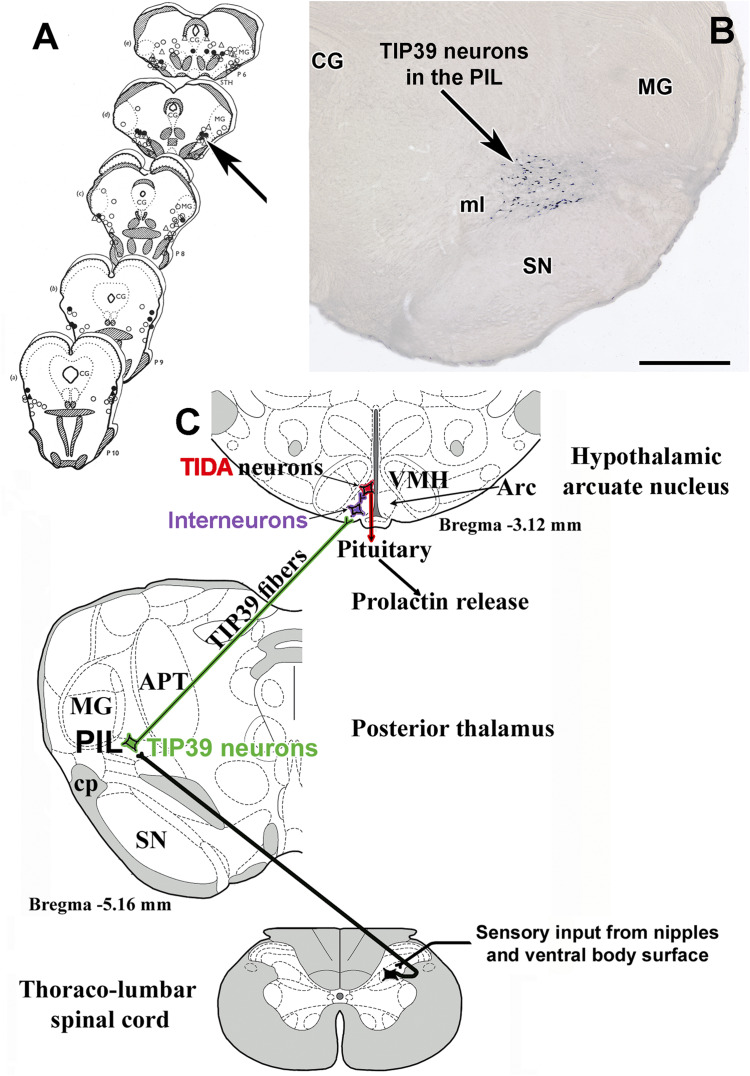
Brain sites whose microstimulation can evoke lactogenesis **(A)** are compared to the position of TIP39 neurons **(B)** in the medial subdivision of the posterior intralaminar complex of the thalamus (PIL). **(C)** The proposed neuronal pathway of suckling-induced prolactin release containing TIP39 neurons in the PIL, which project to the arcuate nucleus. Arrows indicate corresponding brain areas. CG, central gray; MG, medial geniculate body; ml, medial lemniscus; SN, substantia nigra. Scale bar = 1 mm.

The above mentioned, and potentially other mechanisms preventing feedback inhibition of prolactin levels are necessary for elevated prolactin level during lactation. However, the major stimulus that induces prolactin secretion in mothers, is suckling by the pups. Suckling-induced prolactin release was measured in several species. The experimental paradigm most often used in rats includes removal of the pups for 4 h from the dam, during which her serum prolactin levels decrease to a basal minimum level (Neill and Nagy, 1994; Nagy et al., 2005). When the litter is given back to the mother, suckling starts almost immediately. Serum prolactin increases approximately 60 fold, and the maximum level is reached at 30 min following the beginning of suckling (Cservenak et al., 2013). The increased prolactin level is a consequence of reduced dopamine action from the arcuate nucleus (Phillipps et al., 2019). Therefore, tuberoinfundibular dopaminergic neurons must be inhibited by somatosensory stimulus of the nipples. Early studies suggested that this pathway runs ventromedial to the medial geniculate body because microstimulation of this brain area evoked lactogenesis (Tindal and Knaggs, 1969) (Figure 3A). We found neurons expressing tuberoinfundibular peptide of 39 residues (TIP39) in the same location (Dobolyi et al., 2003b) (Figure 3B). TIP39 belongs to the parathyroid hormone family of peptides, which is most abundantly expressed in the brain and is not known to play any role in calcium homeostasis as other members of the peptide family (Suarez-Bregua et al., 2017). Based on previous topographical characterization of the part of the brain expressing TIP39 (Ledoux et al., 1987), we called the position of TIP39 neurons in the medial subdivision of the posterior intralaminar complex of the thalamus (PIL) (Dobolyi et al., 2010). These neurons were activated in response to suckling and markedly increased their TIP39 expression during lactation (Cservenak et al., 2010). Furthermore, the neurons were shown to project to the arcuate nucleus (Dobolyi et al., 2003a), which contains the receptor of TIP39, the parathyroid hormone 2 receptor (PTH2R) (Usdin et al., 2003) in mice (Faber et al., 2007), rat (Dobolyi et al., 2006), and humans (Bago et al., 2009). Injecting a PTH2R antagonist into the lateral ventricle or expressing it with a virus infecting neurons in the vicinity of the arcuate nucleus markedly reduced suckling induced prolactin release suggesting the prolactin-inducing action of TIP39 (Cservenak et al., 2013) (Figure 3C). TIP39 neurons are likely glutamatergic based on electron microscopic and double labeling studies (Cservenak et al., 2017). Glutamate released from these neurons could also be involved in mediating prolactin release. The role of these thalamo-hypothalamic neurons is also consistent with the finding that the pathway, along which projections of TIP39 neurons reach the hypothalamus within the zona incerta (Palkovits et al., 2010), overlaps with the locations of microstimuli evoking lactogenesis rostral to the area ventromedial to the medial geniculate body (Tindal and Knaggs, 1972). Interestingly, stimulation of the preoptic area of the hypothalamus also elicited prolactin secretion. It was interpreted that olfactory information could influence prolactin secretion by that route (Tindal and Knaggs, 1977). In turn, it is also possible that retrograde activation of TIP39 neurons, whose major target is the preoptic area (Cservenak et al., 2017), could also contribute to the stimulatory effect of the preoptic area on prolactin secretion.

The pathway relaying in the PIL (Figure 3C) may also convey the effects of suckling to forebrain sites other than the arcuate nucleus to release prolactin, as suckling induced c-fos expression not only in the PIL (Lin et al., 1998) but also in a variety of different brain regions where PIL neurons project (Li et al., 1999; Lonstein and Stern, 1999). Indeed, the PTH2 receptor was identified in the preoptic area, the paraventricular and dorsomedial hypothalamic nuclei, and the lateral septum (Dobolyi et al., 2006, 2012) and TIP39 terminals were shown to innervate oxytocin neurons in the paraventricular nucleus (Dobolyi et al., 2018) and galanin neurons in the preoptic area (Cservenak et al., 2017) known to control maternal behaviors (Wu et al., 2014).

The functions of prolactin in the postpartum period are numerous. The most well established one is maintaining lactation by acting on mammary epithelial cells. Thereby, prolactin released from a suckling bout enables the mammary gland to further maintain milk production for the next suckling bout via effects of the released prolactin, so in a sense, the pups order their next meal via suckling-induced prolactin release (Phillipps et al., 2019). In addition to lactation, prolactin exerts a variety of different actions in mothers (Bridges and Grattan, 2019) including stimulation of the immune system (Borba et al., 2019) and important effects in the brain by penetrating through the blood-brain barrier (Brown et al., 2016) to reach its multiple targets expressing prolactin receptors in the brain (Bakowska and Morrell, 1997; Kokay et al., 2018) (Figure 2). Prolactin contributes to the increased maternal food intake (Sauve and Woodside, 1996; Naef and Woodside, 2007), lactational anestrus (Grattan and Szawka, 2019), and the induction of maternal behaviors (Brown et al., 2017). These actions are conveyed by the prolactin receptor, although it is established only in some cases which prolactin action is mediated by which location (Table 1). While prolactin is the major maternal hormone affecting the brain in mammals, the brain functions of mothers are also affected by incoming sensory inputs, primarily from the pups. Pups are known to activate a variety of different brain centers, which can be identified at the cellular level using the c-fos technique. The hormonal and neuronal inputs have to support each other to form the proper adaptive responses including maternal behaviors. Their interaction was addressed by double labeling comparing prolactin activated (pSTAT5-positive) versus directly suckling activated (c-fos-positive) brain areas and neurons. Surprisingly, only a relatively small portion of neurons were affected by both stimuli (Olah et al., 2018), suggesting that prolactin provides different types of information for the maternal adaptation of the brain than direct neuronal inputs arriving primarily from the suckling stimulus.

**TABLE 1:** The proposed brain functions of prolactin mediated by prolactin receptors localized in different brain regions.

Brain area	Function	Experimental evidence or *suggested function*
Lateral septal nucleus, ventral (LSv)	Prolactin in LSv may be related to maternal aggression.	*Cabrera-Reyes et al. (2017); Salais-Lopez et al. (2017)*
Anteroventral periventricular nucleus (AVPV)	Dopaminergic neurons expressing pSTAT5 in response to lactation promote maternal care and oxytocin secretion.	Brown et al. (2015); Higo et al. (2015), Scott et al. (2015)
	Lactation induced rapid modulations of kisspeptin are mediated by prolactin.	
Medial preoptic area (MPOA)	Prolactin is necessary for onset of maternal behavior and stimulates maternal care.	Bridges et al. (1990); Wu et al. (2014), Brown et al. (2017); Cservenak et al. (2017)
	Galanin expressing neurons implicated in maternal behavior contain pSTAT5.	
Paraventricular hypothalamic nucleus (PVN)	Oxytocin neurons, involved in lactation and maternal behaviors, express pSTAT5 in lactating rats. Prolactin enhances oxytocin release.	Parker et al. (1991); Nishimori et al. (1996), Fodor et al. (2012); Augustine et al. (2016, 2018)
	Prolactin inhibits vasopressin neurons in lactating rats, which play a part in the development of maternal behavior.	
Arcuate nucleus (Arc)	Dopamine neurons inhibiting prolactin release contain pSTAT5.	Cave et al. (2001); Sapsford et al. (2012), Romano et al. (2013); Araujo-Lopes et al. (2014)
	Prolactin regulates kisspeptin neurons to suppress LH secretion.	
Ventromedial nucleus, ventrolateral (VMHvl)	Activation by prolactin in VMH may be involved in regulation of increased feeding behavior in lactating rats.	*Pi and Grattan (1999)*
Dorsomedial hypothalamic nucleus (DM)	DM regulates food intake and energy balance.	Augustine et al. (2008); Nagaishi et al. (2014), Lopez-Vicchi et al. (2020)
	Prolactin affects DM neurons, which play a role in the metabolic changes triggered by pregnancy and lactation.	
Medial amygdaloid nucleus, posterodorsal (MApd)	Prolactin in MApd may be related to maternal aggression and modulation of the neuroendocrine stress axis.	*Cabrera-Reyes et al. (2017); Salais-Lopez et al. (2017)*
Subparafascicular area (SPF)	not known	
Periaqueductal gray, caudal, ventrolateral (PAGvl)	PAG is critical for suckling induced kyphosis, prolactin may promote it.	*Lonstein and Stern (1997)*
Dorsal raphe nucleus (DR)	Serotonin neurons project to GnRH neurons located in the preoptic area. Prolactin may act on DR serotonin neurons to suppress the activity of GnRH neurons in lactating dams.	*Brown et al. (2011)*
Lateral parabrachial nucleus (LPB)	Not known	
Nucleus of the solitary tract (NTS)	Prolactin plays a role in the metabolic changes triggered by pregnancy and lactation possibly via the NTS, too.	*Brown et al. (2011); Nagaishi et al. (2014)*
	Noradrenergic neurons of NTS origin might mediate the suppression of GnRH neuronal activity.	

*The references written in italics represent only presumed or suggested functions.*

Suckling-induced prolactin release can be prevented by dehydration of the mother. Drinking high salt (2.5%) water for a day reduced suckling-induced prolactin release the following day (Nagy et al., 1992). Acute hyperosmolarity evoked by intraventricular injection of 0.5 ml 10% saline within a suckling bout immediately blocked prolactin release. The blockade of suckling in a hyperosmotic state makes sense as a lactating mother loses a large amount of water during milk production. It is not known whether lactotrophs themselves would be osmoreceptors in mammals; therefore, it is more likely that osmoreceptor cells in the preoptic area convey information on serum osmolarity and blood volume to them, probably via dopaminergic neurons, as both acute and chronic effects of high saline concentrations could be blocked with dopamine receptor antagonists (Nagy et al., 1992). When released, prolactin has anti-diuretic action to replace lost salt and water by nursing. Prolactin can directly act on thirst centers to increase water intake and on renal tubule cells in the kidney to increase salt and water retention (Morrissey et al., 2001), and its indirect action via anti-diuretic hormone (ADH) was also demonstrated (Walker et al., 2001). In addition, ADH could also be increased via the suckling stimulus independent of prolactin (Suzuki et al., 2000).

### Prolactin Released During Incubation in Birds

The serum prolactin level is increased in birds during brooding (Kuwayama et al., 1992), and its level correlated with brooding behavior (Smiley and Adkins-Regan, 2016). In fact, an early prolactin surge is responsible for the formation of a brood patch, a defeathered area on the belly skin, which has an abundant blood supply to effectively transfer heat toward the eggs (Ohkubo, 2017). The role of elevated prolactin is likely to be related to incubation behavior (Smiley, 2019), as prolactin administration can induce brooding behavior (Youngren et al., 1991), while reducing prolactin, e.g., by immunization against it, leads to the cessation of incubation (March et al., 1994). The incubation-promoting effects of prolactin are likely to be mediated via prolactin receptors in the preoptic area (Youngren et al., 1989). In addition, prolactin may also contribute to the increased aggressive and defensive behaviors of incubating birds (Romanov et al., 2002) and to the decline in gonadal function by inhibiting gonadotropin-releasing hormone-producing neurons (Rozenboim et al., 1993). Our knowledge of the time course of changes in prolactin levels and actions in the incubation period is not good. It would be interesting to know how fast the prolactin level decreases if the parent is removed from the nest and how fast prolactin rises when allowed to incubate again.

The mechanism by which prolactin is induced by incubation or for incubation is not fully understood. In birds, dopamine can inhibit spontaneous prolactin release from lactotrophs via D2 receptors (Christensen and Vleck, 2008), similar to mammals. However, dopamine also has a stimulatory action via D1 receptors best established in the turkey (Bhatt et al., 2003; Chaiseha et al., 2003) but also in other avian species (Xu et al., 2010). Action via D1 receptors can stimulate hypothalamic neurons expressing VIP (Sartsoongnoen et al., 2008), which acts as a prolactin-releasing hormone in avian species (Proudman and Opel, 1988; Kosonsiriluk et al., 2008). It is unlikely that sexual hormones elicit prolactin release during incubation because increased prolactin levels are found even in ovariectomized birds during incubation. Thus, it is plausible that somatosensory input from the eggs itself can evoke prolactin secretion via neuronal pathways (Massaro et al., 2007). Indeed, swapping eggs from the parents leads to reduced serum prolactin levels (Sinpru et al., 2018). The potential neuronal pathways involved have not been revealed to date. It is not possible that TIP39 plays a role in prolactin release in avian species as it does in mammals because the gene encoding this peptide is missing in birds, even though it is present in all other vertebrate taxa (On et al., 2015), but a homologous neuronal pathway could be involved. Furthermore, it would be interesting to learn whether osmolarity affects prolactin release in birds during incubation as it does during lactation in mammals. However, this question has not been addressed to date, even though it is reasonable that dehydration contributes to the cessation of actual incubation driven by both thirst and a reduced parental motivation due to reduced prolactin level.

In addition to brooding behavior, many avian species, the altricial birds, also show parental behavior in the form of feeding the nestlings (regurgitation) in the posthatching period. Since prolactin level is not particularly high in the posthatching period, feeding behavior may not depend on prolactin in most avian species even though the reduction of prolactin following hatching is slower in altricial as compared to precocial birds (Lea and Sharp, 1991) and prolactin level correlated with individual differences in parental behavior in the passerine zebra finch (Smiley and Adkins-Regan, 2016). In turn, rearing behavior in the posthatching period may be induced by tactile stimulation by nestlings in combination with visual or auditory inputs (Richard-Yris et al., 1998). These inputs are likely mediated toward lactotrophs for prolactin secretion by VIP neurons in the arcuate nucleus as during incubation (Buntin et al., 1991). Tactile, visual and auditory inputs lead to direct neuronal activation of parental brain centers in zebra finch (Fazekas et al., 2020). To date, the interconnection of these activated cells with those sensitive to prolactin has not been established in birds as in mice (Olah et al., 2018), despite the availability of pSTAT5 immunolabeling in birds (Buntin and Buntin, 2014). In addition to a presumed role of prolactin in parenting behavior in birds (Buntin et al., 1991), the hormone may have an additional, specific role in avian species that produce crop milk (Wan et al., 2019). In fact, prolactin was originally discovered by its ability to induce crop milk production in the pigeons. Still, the mechanism of prolactin secretion for crop milk production has not yet been established. Microstimulation experiments suggested that the preoptic area may be involved in crop milk production while direct stimulation of the mediobasal hypothalamus including the median eminence, had no effect (Kanematsu, 1980).

### The Roles of Prolactin in Fish Parenting

Parental behaviors appear in about 30% of teleost fish (Royle et al., 2014). Interestingly, in these species, the males provide parental care somewhat more often than the females (Reynolds et al., 2002). Although parental behaviors have various forms in fish, the most common forms include nest building and egg (or embryo) attendance. The latter not only protects the eggs from predators but fanning also aerates the eggs and frees them from debris (Rosenblatt, 2003). Although the available knowledge on the control of these behaviors is scarce, some recent data indicated the potential involvement of prolactin (Whittington and Wilson, 2013). Prolactin implants increased nurturing behavior in bluegill (*Lepomis macrochirus*) (Cunha et al., 2019) and also in three-spined stickleback (*Gasterosteus aculeatus*) (de Ruiter et al., 1986). Prolactin level may rise during spawning and remains elevated during parental care in cichlid fish (*Oreochromis niloticus)* (Tacon et al., 2000). The mechanism how prolactin in induced in unknown at present although simple cues as sensory inputs from the eggs are a likely candidate for fanning (Dulac et al., 2014). It should also be mentioned that data arguing against a role of prolactin in fish parenting are also available (Bender et al., 2008).

## Osmoregulatory Actions of Prolactin

Osmoregulation is a complex process that includes a variety of different regulatory hormones and systems including prolactin but also renin-angiotensin, anti-diuretic hormone, aldosterone, and atrial natriuretic peptide (Takei et al., 2014). These endocrine components of the regulatory system are relatively conserved in vertebrates despite the differing needs of various animals (the major difference between fish and mammals being the lack of involvement of aldosterone in fish), while the neuronal components of osmoregulation show remarkable differences. Notably, in mammals, forebrain circumventricular organs, such as the vascular organ of the lamina terminalis and the subfornical organ play pivotal roles in sensing both plasma osmolarity and plasma hormone content, most importantly angiotensin II levels (McKinley and Johnson, 2004). These organs convey this information toward thirst centers of the cerebral cortex as well as toward neurohypophyseal neurons synthesizing antidiuretic hormone (ADH). ADH neurons are themselves osmosensors, which secrete ADH in response to hyperosmolarity (Bourque and Oliet, 1997). Teleost fish do not have a subfornical organ and the role of the vascular organ of the lamina terminalis is not known in osmoregulation (Katayama et al., 2018). They may be able to respond to angiotensin II detected through the area a postrema (Nobata et al., 2013), a circumventricular organ in the hindbrain. However, the major regulator of their drinking in vagal input from peripheral receptors (Mayer-Gostan and Hirano, 1976). These differences between clades may simply is a consequence of forebrain development in mammals, which better allows reconciliation of drinking with other behaviors. Alternatively, the different requirement associated terrestrial environment created the new type of osmoregulatory systems. Indeed, terrestrial animals need to retain water while fish have to deal with the difference in their ionic composition from their environment with which they are in direct contact. Generally, the environment of fish can be freshwater or saltwater, which requires very different regulations. Furthermore, there are euryhaline species that can live in both fresh and seawater, as they migrate or live in brackish water. The major difference between fresh and seawater adaptation of fish is that in the gills, they actively excrete salt in seawater while actively take up salt in fresh water. In addition, fish drink much more in seawater than in fresh water (Takei et al., 2014). Prolactin was shown to play a role in freshwater adaptation as first demonstrated in killifish (*Fundulus heteroclitus*), a species that could survive following hypophysectomy in fresh water only in the presence of external prolactin (Pickford and Phillips, 1959). More recently, strong evidence came from zebrafish models lacking the prolactin gene: the larvae survived to adulthood in brackish but not in egg water (Shu et al., 2016). This role of prolactin is widespread in euryhaline fish but not ubiquitous, as catfish and salmonids can survive in fresh water following hypophysectomy without prolactin (Hirano, 1986). Although it is possible that the latter species produce prolactin outside of the pituitary as lactotrophs could be located in other locations in relatively primitive eels (Sakamoto and McCormick, 2006). Nevertheless, all teleost species have pituitary with lactotrophs in their anterior lobe; therefore, it is more likely that other osmoregulatory systems can provide freshwater adaptability in these species.

Prolactin exerts its regulatory function in all branchial epithelia, including the gill, kidney, urinary bladder, and gastrointestinal tract (Takei et al., 2014). In fish, the major osmoregulatory organ is the gill where ionocytes are located for water and ion transport while the kidney has the most important osmoregulatory function in mammals (Manzon, 2002). Prolactin may be able induce the proliferation of specific types of ionocytes, which can remove water and take up ions (Hiroi and McCormick, 2012). While prolactin is among the osmoregulatory hormones with relatively slower actions in general, it is also able to induce and stimulate ion transporters, such as Na^+^/Cl^–^ cotransporter and the Na^+^/K^+^ pump (Breves et al., 2014) via its receptors expressed in ionocytes (Santos et al., 2001). It is also under investigation how the 2 types of prolactin receptors (a and b) both expressed in osmoregulatory epithelia of several teleost species support each other’s actions. Less evidence is available but it is still likely that prolactin can inhibit aquaporins, e.g., aquaporin 3, which is known to be involved in freshwater adaptation (Lignot et al., 2002; Breves et al., 2016), and induce tight junction forming cadherins to reduce water uptake in the gills. Our knowledge is more limited on potential action of prolactin on teleost kidney, bladder or inhibition of drinking, which are all potential sites of action. Furthermore, the osmoregulatory function of prolactin is less pronounced in mammals unless we consider its effect on milk production as an osmoregulatory action. Milk contains high amount of fluid as well as sodium ion. Old literature suggested water retention ability of prolactin, however, it was shown to be caused by contamination with ADH (Keeler and Wilson, 1976). More recent experiments demonstrated increased sodium and chloride retention by prolactin (Greenlee et al., 2015) while natriuretic effect in mammals by acting on the Na^+^/K^+^ pump via local dopaminergic system in the proximal renal tubules has also been reported (Ibarra et al., 2005). The effect of prolactin on the kidney function of birds is also not known. However, an interesting stimulatory effect of prolactin has been reported on the duck nasal salt gland, an important avian salt excretory organ (Peaker et al., 1970).

The secretion of prolactin in response to changes in osmolarity is less well studied than its osmoregulatory actions. Nevertheless, it has been demonstrated that hyposmolarity evokes prolactin release from the pituitary of teleost fish and an increase in prolactin gene expression also takes place (Lee et al., 2006; Fuentes et al., 2010). It seems likely that lactotrophs themselves are osmosensitive in fish and release prolactin in response to the hypoosmotic local environment (Kwong et al., 2009; Watanabe et al., 2009; Seale et al., 2012). Furthermore, the prolactin secretion as an osmotic response is not affected by pharmacological blockade of dopamine receptors (Liu et al., 2006). Evidence is available that cultured lactotrophs react to reduced osmolarity by prolactin secretion and that it is accompanied by increased cell size of the lactotrophs (Weber et al., 2004). In lactotrophs from Tilapia, the increased cell volume was blocked by aquaporin inhibitors (Watanabe et al., 2009). Given that aquaporin 3 is present in Tilapia lactotrophs (Watanabe et al., 2005), its involvement in water intake of the cells is likely. The increased volume may activate the transient receptor potential-vanilloid (TRPV) 4 receptor, a stretch-activated calcium channel (Watanabe et al., 2002), and the resulting elevated intracellular calcium level leads to prolactin release (Seale et al., 2012). A positive feedback of prolactin has also been suggested to increase the response (Yamaguchi et al., 2016). For long-term adaptation to fresh water, prolactin expression in tremendously increased while aquaporin 3 and TRPV 4 expression are reduced for sensitization of prolactin secretion (Seale et al., 2012). In contrast to fish, there is no compelling evidence on the direct osmosensitivity of lactotrophs in mammals or in other vertebrate taxa. The mammals have osmosensitive cells in the vascular organ of the lamina terminalis and the subfornical organ (McKinley and Johnson, 2004). Neuronal output from these preoptic hypothalamic regions can reach vasopressin neurons in the paraventricular nucleus and thirst centers of the brain. It seems likely that dopaminergic neurons in the mediobasal hypothalamus regulating prolactin secretion from lactotrophs also receive information on osmolarity from the same preoptic receptor cells as hyperosmolarity prevented suckling-induced prolactin release but dopamine receptor antagonists could block the effect of hypoosmolarity as discussed above (Nagy et al., 1992). On the other hand, it has not been addressed in detail whether other types of prolactin release, e.g., during pregnancy or stress can also be inhibited by hyperosmolarity in the pituitary in tetrapods although different effects of osmotic inhibition on differently elicited prolactin release have been reported (Dohanics et al., 1994). It is also not known if prolactin release from mammalian lactotrophs are influenced by osmolality of its environment physiologically. Some early results indicated direct osmoreceptive lactotrophs in the mammalian pituitary (Labella et al., 1975; Lorenson and Jacobs, 1987). More recently, it was found that sub-physiological hypotonicity elicited transient release followed by sustained depression of prolactin release from perfused rat lactotrophs (Jorgacevski et al., 2008). The role of osmolality in prolactin secretion of birds is also not established even though it would also be interesting to address whether prolactin secretion during incubation in birds can be prevented by hyperosmolarity. It would make sense if the bird parent would stop incubating and go drinking when hyperosmolarity occurs.

## Conclusion

**FIGURE 4:**
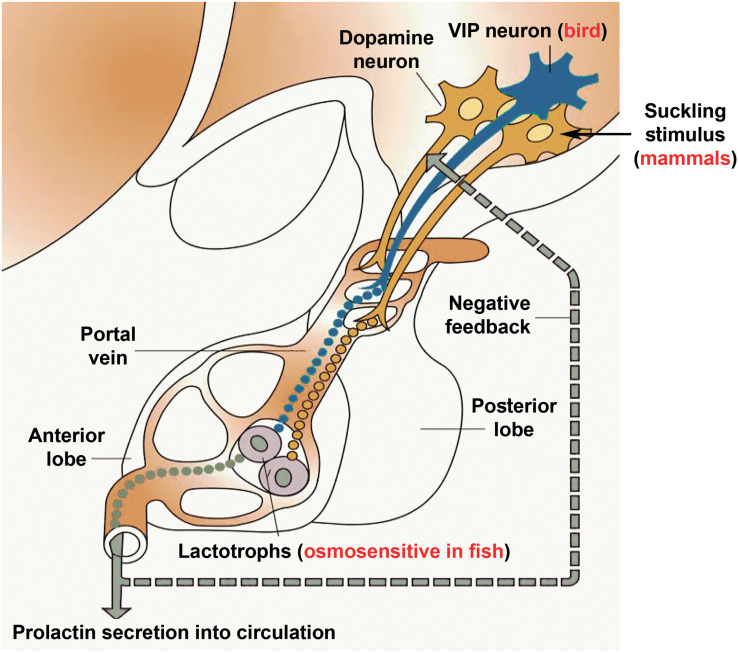
Schematics on the control of prolactin secretion. Prolactin releasing factor, which is truly established only in birds to be vasoactive intestinal polypeptide (VIP), reaches the anterior lobe of the pituitary via the portal circulation to evoke prolactin secretion from the prolactin-producing lactotrophs located in the pituitary. In contrast, prolactin release in fish is most profoundly driven by hyposmolarity sensed by the lactotrophs themselves. In contrast, mammalian lactotrophs release prolactin spontaneously, and the regulation takes place by inhibition, which is carried out by dopamine released from dopaminergic neurons located in the mediobasal hypothalamus. The inhibition of prolactin secretion by dopamine is likely present in all vertebrate taxa. In turn, suckling-induced prolactin secretion is characteristic of mammals only, in which suckling stimulus of the pups is conveyed to dopaminergic neurons in a classic neuroendocrine reflex to stimulate prolactin release according to the need of the pups. The clade-specific features were added to a modified version of the basic scheme (Loìpez et al., 2003).

Prolactin is a vertebrate-specific hormone whose functions have been studied for a long time. Indeed, immense knowledge has accumulated on prolactin secretion and function in a variety of different species. Thus, prolactin represents an exciting opportunity for evolutionary neuroendocrinology as its functions are compared between the different species and even different classes of vertebrates. For example, prolactin, a major osmoregulator in fish turned into a hormone that regulates lactation in mammals. In fact, both of these functions require the action of prolactin on epithelial cells, both as far as their proliferation and the control of their transport processes. Another intriguing change is the parental behavioral action of prolactin, which already appears in fish (Cunha et al., 2019) but becomes prominent in birds and mammals. In both classes, parental care has different forms, such as brooding and nursing behaviors. Other effects of prolactin, such as inhibiting gonadotropins is also maintained in a variety of different taxa. Evolutionary comparison of the regulation of prolactin secretion is also instructive. Direct osmosensitivity of lactotrophs is characteristic only of fish; however, hyperosmolarity also inhibits prolactin release in mammals, and research investigating this question is also proposed in birds. The inhibitory influence of dopamine of hypothalamic origin on prolactin secretion seems to be present in all taxa although its role is most important in mammals in which a regulatory releasing mechanism has not been identified to date (Figure 4).

## Author Contributions

All authors participated in the design of literature review, reading of relevant manuscripts, interpretation of the literature, and writing of the manuscript.

## Conflict of Interest

The authors declare that the research was conducted in the absence of any commercial or financial relationships that could be construed as a potential conflict of interest.

## References

[B1] Andrews Z. B. Grattan D. R. (2003). Opioid receptor subtypes involved in the regulation of prolactin secretion during pregnancy and lactation. *J. Neuroendocrinol.* 15 227–236. 10.1046/j.1365-2826.2003.00975.x 12588510

[B2] Aoki M. Wartenberg P. Grunewald R. Phillipps H. R. Wyatt A. Grattan D. R. (2019). Widespread cell-specific prolactin receptor expression in multiple murine organs. *Endocrinology* 160 2587–2599. 10.1210/en.2019-00234 31373638

[B3] Araujo-Lopes R. Crampton J. R. Aquino N. S. Miranda R. M. Kokay I. C. Reis A. M. (2014). Prolactin regulates kisspeptin neurons in the arcuate nucleus to suppress lh secretion in female rats. *Endocrinology* 155 1010–1020. 10.1210/en.2013-1889 24456164

[B4] Arbogast L. A. Voogt J. L. (1995). Hypoprolactinemia decreases tyrosine hydroxylase activity in the tuberoinfundibular dopaminergic neurons acutely by protein dephosphorylation and chronically by changes in gene expression. *Endocrine* 3 801–806. 10.1007/bf02935684 21153124

[B5] Augustine R. A. Bouwer G. T. Seymour A. J. Grattan D. R. Brown C. H. (2016). Reproductive regulation of gene expression in the hypothalamic supraoptic and paraventricular nuclei. *J. Neuroendocrinol.* 28 1–12. 10.1111/jne.12350 26670189

[B6] Augustine R. A. Ladyman S. R. Grattan D. R. (2008). From feeding one to feeding many: hormone-induced changes in bodyweight homeostasis during pregnancy. *J. Physiol.* 586 387–397. 10.1113/jphysiol.2007.146316 18033810 PMC2375600

[B7] Augustine R. A. Seymour A. J. Campbell R. E. Grattan D. R. Brown C. H. (2018). Integrative neuro-humoral regulation of oxytocin neuron activity in pregnancy and lactation. *J. Neuroendocrinol.* 30 1–15. 10.1111/jne.12569 29323764

[B8] Auperin B. Rentier-Delrue F. Martial J. A. Prunet P. (1995). Regulation of gill prolactin receptors in tilapia (*Oreochromis niloticus*) after a change in salinity or hypophysectomy. *J. Endocrinol.* 145 213–220. 10.1677/joe.0.1450213 7616154

[B9] Auriemma R. S. Pirchio R. De Alcubierre D. Pivonello R. Colao A. (2019). Dopamine agonists: from the 1970s to today. *Neuroendocrinology* 109 34–41. 10.1159/000499470 30852578

[B10] Bago A. G. Dimitrov E. Saunders R. Seress L. Palkovits M. Usdin T. B. (2009). Parathyroid hormone 2 receptor and its endogenous ligand tuberoinfundibular peptide of 39 residues are concentrated in endocrine, viscerosensory and auditory brain regions in macaque and human. *Neuroscience* 162 128–147. 10.1016/j.neuroscience.2009.04.054 19401215 PMC2703999

[B11] Bakowska J. C. Morrell J. I. (1997). Atlas of the neurons that express mrna for the long form of the prolactin receptor in the forebrain of the female rat. *J. Comp. Neurol.* 386 161–177. 10.1002/(sici)1096-9861(19970922)386:2<161::aid-cne1>3.0.co;2-# 9295145

[B12] Bakowska J. C. Morrell J. I. (2003). The distribution of mrna for the short form of the prolactin receptor in the forebrain of the female rat. *Brain Res. Mol. Brain Res.* 116 50–58. 10.1016/s0169-328x(03)00213-4 12941460

[B13] Bender N. Taborsky M. Power D. M. (2008). The role of prolactin in the regulation of brood care in the cooperatively breeding fish neolamprologus pulcher. *J. Exp. Zool. A Ecol. Genet. Physiol.* 309 515–524. 10.1002/jez.482 18663725

[B14] Bern H. A. Nicoll C. S. (1968). The comparative endocrinology of prolactin. *Recent Prog Horm Res* 24 681–720. 4882330 10.1016/b978-1-4831-9827-9.50019-8

[B15] Bhatt R. Youngren O. Kang S. El Halawani M. (2003). Dopamine infusion into the third ventricle increases gene expression of hypothalamic vasoactive intestinal peptide and pituitary prolactin and luteinizing hormone beta subunit in the turkey. *Gen. Comp. Endocrinol.* 130 41–47. 10.1016/s0016-6480(02)00533-6 12535623

[B16] Bjoro T. Sand O. Ostberg B. C. Gordeladze J. O. Torjesen P. Gautvik K. M. (1990). The mechanisms by which vasoactive intestinal peptide (vip) and thyrotropin releasing hormone (trh) stimulate prolactin release from pituitary cells. *Biosci. Rep.* 10 189–199. 10.1007/bf01116578 2162702

[B17] Bole-Feysot C. Goffin V. Edery M. Binart N. Kelly P. A. (1998). Prolactin (prl) and its receptor: actions, signal transduction pathways and phenotypes observed in prl receptor knockout mice. *Endocr. Rev.* 19 225–268. 10.1210/edrv.19.3.0334 9626554

[B18] Borba V. V. Zandman-Goddard G. Shoenfeld Y. (2019). Prolactin and autoimmunity: the hormone as an inflammatory cytokine. *Best Pract. Res. Clin. Endocrinol. Metab.* 33:101324. 10.1016/j.beem.2019.101324 31564625

[B19] Bourque C. W. Oliet S. H. (1997). Osmoreceptors in the central nervous system. *Annu. Rev. Physiol.* 59 601–619. 10.1146/annurev.physiol.59.1.601 9074779

[B20] Breves J. P. Inokuchi M. Yamaguchi Y. Seale A. P. Hunt B. L. Watanabe S. (2016). Hormonal regulation of aquaporin 3: opposing actions of prolactin and cortisol in tilapia gill. *J. Endocrinol.* 230 325–337. 10.1530/joe-16-0162 27402066

[B21] Breves J. P. McCormick S. D. Karlstrom R. O. (2014). Prolactin and teleost ionocytes: new insights into cellular and molecular targets of prolactin in vertebrate epithelia. *Gen. Comp. Endocrinol.* 203 21–28. 10.1016/j.ygcen.2013.12.014 24434597 PMC4096611

[B22] Bridges R. S. Grattan D. R. (2019). 30 years after: CNS actions of prolactin: sources, mechanisms and physiological significance. *J. Neuroendocrinol.* 31:e12669. 10.1111/jne.12669 30549349

[B23] Bridges R. S. Numan M. Ronsheim P. M. Mann P. E. Lupini C. E. (1990). Central prolactin infusions stimulate maternal behavior in steroid-treated, nulliparous female rats. *Proc. Natl. Acad. Sci. U.S.A.* 87 8003–8007. 10.1073/pnas.87.20.8003 2236014 PMC54880

[B24] Bridges R. S. Robertson M. C. Shiu R. P. C. Friesen H. G. Stuer A. M. Mann P. E. (1996). Endocrine communication between conceptus and mother: placental lactogen stimulation of maternal behavior. *Neuroendocrinology* 64 57–64. 10.1159/000127098 8811667

[B25] Brinca L. Fuentes J. Power D. M. (2003). The regulatory action of estrogen and vasoactive intestinal peptide on prolactin secretion in sea bream (sparus aurata, l.). *Gen. Comp. Endocrinol.* 131 117–125. 10.1016/s0016-6480(02)00628-7 12679088

[B26] Brown R. S. Herbison A. E. Grattan D. R. (2011). Differential changes in responses of hypothalamic and brainstem neuronal populations to prolactin during lactation in the mouse. *Biol. Reprod.* 84 826–836. 10.1095/biolreprod.110.089185 21178171

[B27] Brown R. S. Herbison A. E. Grattan D. R. (2015). Effects of prolactin and lactation on a15 dopamine neurones in the rostral preoptic area of female mice. *J. Neuroendocrinol.* 27 708–717. 10.1111/jne.12297 26132331

[B28] Brown R. S. Kokay I. C. Herbison A. E. Grattan D. R. (2010). Distribution of prolactin-responsive neurons in the mouse forebrain. *J. Comp. Neurol.* 518 92–102. 10.1002/cne.22208 19882722

[B29] Brown R. S. Piet R. Herbison A. E. Grattan D. R. (2012). Differential actions of prolactin on electrical activity and intracellular signal transduction in hypothalamic neurons. *Endocrinology* 153 2375–2384. 10.1210/en.2011-2005 22416085

[B30] Brown R. S. Wyatt A. K. Herbison R. E. Knowles P. J. Ladyman S. R. Binart N. (2016). Prolactin transport into mouse brain is independent of prolactin receptor. *FASEB J.* 30 1202–1210. 10.1096/fj.15-276519 26567005

[B31] Brown R. S. E. Aoki M. Ladyman S. R. Phillipps H. R. Wyatt A. Boehm U. (2017). Prolactin action in the medial preoptic area is necessary for postpartum maternal nursing behavior. *Proc. Natl. Acad. Sci. U.S.A.* 114 10779–10784. 10.1073/pnas.1708025114 28923971 PMC5635892

[B32] Bu G. Liang X. Li J. Wang Y. (2015). Extra-pituitary prolactin (prl) and prolactin-like protein (prl-l) in chickens and zebrafish. *Gen. Comp. Endocrinol.* 220 143–153. 10.1016/j.ygcen.2015.02.001 25683198

[B33] Buntin J. D. Becker G. M. Ruzycki E. (1991). Facilitation of parental behavior in ring doves by systemic or intracranial injections of prolactin. *Horm. Behav.* 25 424–444. 10.1016/0018-506x(91)90012-7 1937430

[B34] Buntin J. D. Buntin L. (2014). Increased stat5 signaling in the ring dove brain in response to prolactin administration and spontaneous elevations in prolactin during the breeding cycle. *Gen. Comp. Endocrinol.* 200 1–9. 10.1016/j.ygcen.2014.02.006 24530808 PMC3995851

[B35] Cabrera-Reyes E. A. Limon-Morales O. Rivero-Segura N. A. Camacho-Arroyo I. Cerbon M. (2017). Prolactin function and putative expression in the brain. *Endocrine* 57 199–213. 10.1007/s12020-017-1346-x 28634745

[B36] Campbell G. S. Argetsinger L. S. Ihle J. N. Kelly P. A. Rillema J. A. Carter-Su C. (1994). Activation of jak2 tyrosine kinase by prolactin receptors in nb2 cells and mouse mammary gland explants. *Proc. Natl. Acad. Sci. U.S.A.* 91 5232–5236. 10.1073/pnas.91.12.5232 7515493 PMC43968

[B37] Carretero J. Angoso M. Rubio M. Blanco E. J. Sierra E. Herrero J. J. (2006). In vitro immunoblockade of vip inhibits the proliferation of pituitary prolactin cells. *Anat. Embryol. (Berl.)* 211 11–18. 10.1007/s00429-005-0058-9 16328361

[B38] Cave B. J. Wakerley J. B. Luckman S. M. Tortonese D. J. (2001). Hypothalamic targets for prolactin: assessment of c-fos induction in tyrosine hydroxylase- and proopiomelanocortin-containing neurones in the rat arcuate nucleus following acute central prolactin administration. *Neuroendocrinology* 74 386–395. 10.1159/000054705 11752895

[B39] Chaiseha Y. Youngren O. Al-Zailaie K. El Halawani M. (2003). Expression of d1 and d2 dopamine receptors in the hypothalamus and pituitary during the turkey reproductive cycle: colocalization with vasoactive intestinal peptide. *Neuroendocrinology* 77 105–118. 10.1159/000068649 12624532

[B40] Christensen D. Vleck C. M. (2008). Prolactin release and response to vasoactive intestinal peptide in an opportunistic breeder, the zebra finch (*Taeniopygia guttata*). *Gen. Comp. Endocrinol.* 157 91–98. 10.1016/j.ygcen.2008.04.013 18555065

[B41] Christian H. C. Chapman L. P. Morris J. F. (2007). Thyrotrophin-releasing hormone, vasoactive intestinal peptide, prolactin-releasing peptide and dopamine regulation of prolactin secretion by different lactotroph morphological subtypes in the rat. *J. Neuroendocrinol.* 19 605–613. 10.1111/j.1365-2826.2007.01567.x 17620102

[B42] Clevenger C. V. Kline J. B. (2001). Prolactin receptor signal transduction. *Lupus* 10 706–718. 10.1191/096120301717164949 11721697

[B43] Crowley W. R. (2015). Neuroendocrine regulation of lactation and milk production. *Compr. Physiol.* 5 255–291. 10.1002/cphy.c140029 25589271

[B44] Cservenak M. Bodnar I. Usdin T. B. Palkovits M. Nagy G. M. Dobolyi A. (2010). Tuberoinfundibular peptide of 39 residues is activated during lactation and participates in the suckling-induced prolactin release in rat. *Endocrinology* 151 5830–5840. 10.1210/en.2010-0767 20861230 PMC2999487

[B45] Cservenak M. Kis V. Keller D. Dimen D. Menyhart L. Olah S. (2017). Maternally involved galanin neurons in the preoptic area of the rat. *Brain Struct. Funct.* 222 781–798. 10.1007/s00429-016-1246-5 27300187 PMC5156581

[B46] Cservenak M. Szabo E. R. Bodnar I. Leko A. Palkovits M. Nagy G. M. (2013). Thalamic neuropeptide mediating the effects of nursing on lactation and maternal motivation. *Psychoneuroendocrinology* 38 3070–3084. 10.1016/j.psyneuen.2013.09.004 24094875 PMC3844093

[B47] Cunha A. A. P. Partridge C. G. Knapp R. Neff B. D. (2019). Androgen and prolactin manipulation induces changes in aggressive and nurturing behavior in a fish with male parental care. *Horm. Behav.* 116:104582. 10.1016/j.yhbeh.2019.104582 31445012

[B48] de Ruiter A. J. Wendelaar Bonga S. E. Slijkhuis H. Baggerman B. (1986). The effect of prolactin on fanning behavior in the male three-spined stickleback, gasterosteus aculeatus l. *Gen. Comp. Endocrinol.* 64 273–283. 10.1016/0016-6480(86)90014-6 3557093

[B49] Dobolyi A. Cservenak M. Young L. J. (2018). Thalamic integration of social stimuli regulating parental behavior and the oxytocin system. *Front. Neuroendocrinol.* 51:2. 10.1016/j.yfrne.2018.05.002 29842887 PMC6175608

[B50] Dobolyi A. Dimitrov E. Palkovits M. Usdin T. B. (2012). The neuroendocrine functions of the parathyroid hormone 2 receptor. *Front. Endocrinol. (Lausanne)* 3:121. 10.3389/fendo.2012.00121 23060860 PMC3465808

[B51] Dobolyi A. Irwin S. Wang J. Usdin T. B. (2006). The distribution and neurochemistry of the parathyroid hormone 2 receptor in the rat hypothalamus. *Neurochem. Res.* 31 227–236. 10.1007/s11064-005-9011-9 16570212

[B52] Dobolyi A. Leko A. H. (2019). The insulin-like growth factor-1 system in the adult mammalian brain and its implications in central maternal adaptation. *Front. Neuroendocrinol.* 52:2. 10.1016/j.yfrne.2018.12.002 30552909

[B53] Dobolyi A. Palkovits M. Bodnar I. Usdin T. B. (2003a). Neurons containing tuberoinfundibular peptide of 39 residues project to limbic, endocrine, auditory and spinal areas in rat. *Neuroscience* 122 1093–1105. 10.1016/j.neuroscience.2003.08.034 14643775

[B54] Dobolyi A. Palkovits M. Usdin T. B. (2003b). Expression and distribution of tuberoinfundibular peptide of 39 residues in the rat central nervous system. *J. Comp. Neurol.* 455 547–566. 10.1002/cne.10515 12508326

[B55] Dobolyi A. Palkovits M. Usdin T. B. (2010). The tip39-pth2 receptor system: unique peptidergic cell groups in the brainstem and their interactions with central regulatory mechanisms. *Prog. Neurobiol.* 90 29–59. 10.1016/j.pneurobio.2009.10.017 19857544 PMC2815138

[B56] Dohanics J. Smith M. S. Blackburn R. E. Verbalis J. G. (1994). Osmotic inhibition of prolactin secretion in rats. *J. Neuroendocrinol.* 6 291–298. 10.1111/j.1365-2826.1994.tb00585.x 7920595

[B57] Dulac C. O’Connell L. A. Wu Z. (2014). Neural control of maternal and paternal behaviors. *Science* 345 765–770. 10.1126/science.1253291 25124430 PMC4230532

[B58] Egli M. Bertram R. Sellix M. T. Freeman M. E. (2004). Rhythmic secretion of prolactin in rats: action of oxytocin coordinated by vasoactive intestinal polypeptide of suprachiasmatic nucleus origin. *Endocrinology* 145 3386–3394. 10.1210/en.2003-1710 15033917 PMC1993890

[B59] Everett J. W. (1964). Central neural control of reproductive functions of the adenohypophysis. *Physiol. Rev.* 44 373–431. 10.1152/physrev.1964.44.3.373 14175665

[B60] Exton M. S. Kruger T. H. Koch M. Paulson E. Knapp W. Hartmann U. (2001). Coitus-induced orgasm stimulates prolactin secretion in healthy subjects. *Psychoneuroendocrinology* 26 287–294. 10.1016/s0306-4530(00)00053-6 11166491

[B61] Faber C. A. Dobolyi A. Sleeman M. Usdin T. B. (2007). Distribution of tuberoinfundibular peptide of 39 residues and its receptor, parathyroid hormone 2 receptor, in the mouse brain. *J. Comp. Neurol.* 502 563–583. 10.1002/cne.21330 17394159 PMC2923585

[B62] Fazekas E. A. Morvai B. Zachar G. Dora F. Szekely T. Pogany A. (2020). Neuronal activation in zebra finch parents associated with reintroduction of nestlings. *J. Comp. Neurol.* 528 363–379. 10.1002/cne.24761 31423585

[B63] Fernandez-Valdivia R. Mukherjee A. Ying Y. Li J. Paquet M. DeMayo F. J. (2009). The rankl signaling axis is sufficient to elicit ductal side-branching and alveologenesis in the mammary gland of the virgin mouse. *Dev. Biol.* 328 127–139. 10.1016/j.ydbio.2009.01.019 19298785

[B64] Fodor A. Klausz B. Pinter O. Daviu N. Rabasa C. Rotllant D. (2012). Maternal neglect with reduced depressive-like behavior and blunted c-fos activation in brattleboro mothers, the role of central vasopressin. *Horm. Behav.* 62 539–551. 10.1016/j.yhbeh.2012.09.003 23006866

[B65] Freeman M. E. Kanyicska B. Lerant A. Nagy G. (2000). Prolactin: structure, function, and regulation of secretion. *Physiol. Rev.* 80 1523–1631. 10.1152/physrev.2000.80.4.1523 11015620

[B66] Fuentes J. Brinca L. Guerreiro P. M. Power D. M. (2010). Prl and gh synthesis and release from the sea bream (*Sparus auratus* l.) pituitary gland in vitro in response to osmotic challenge. *Gen. Comp. Endocrinol.* 168 95–102. 10.1016/j.ygcen.2010.04.005 20406642

[B67] Goffin V. Shiverick K. T. Kelly P. A. Martial J. A. (1996). Sequence-function relationships within the expanding family of prolactin, growth hormone, placental lactogen, and related proteins in mammals. *Endocr. Rev.* 17 385–410. 10.1210/er.17.4.385 8854051

[B68] Gonzalez-Iglesias A. E. Murano T. Li S. Tomic M. Stojilkovic S. S. (2008). Dopamine inhibits basal prolactin release in pituitary lactotrophs through pertussis toxin-sensitive and -insensitive signaling pathways. *Endocrinology* 149 1470–1479. 10.1210/en.2007-0980 18096663 PMC2276716

[B69] Grattan D. R. (2015). The hypothalamo-prolactin axis. *J. Endocrinol.* 226 T101–T122. 26101377 10.1530/JOE-15-0213 PMC4515538

[B70] Grattan D. R. Szawka R. E. (2019). Kisspeptin and prolactin. *Semin. Reprod. Med.* 37 93–104. 31847029 10.1055/s-0039-3400956

[B71] Greenlee M. M. Mitzelfelt J. D. Duke B. J. Al-Khalili O. Bao H. F. Eaton D. C. (2015). Prolactin stimulates sodium and chloride ion channels in a6 renal epithelial cells. *Am. J. Physiol. Renal Physiol.* 308 F697–F705. 25587116 10.1152/ajprenal.00270.2014 PMC4385885

[B72] Gunnet J. W. Freeman M. E. (1983). The mating-induced release of prolactin: a unique neuroendocrine response. *Endocr. Rev.* 4 44–61. 10.1210/edrv-4-1-44 6339213

[B73] Gunnet J. W. Freeman M. E. (1984). Hypothalamic regulation of mating-induced prolactin release. Effect of electrical stimulation of the medial preoptic area in conscious female rats. *Neuroendocrinology* 38 12–16. 10.1159/000123859 6537996

[B74] Higo S. Aikawa S. Iijima N. Ozawa H. (2015). Rapid modulation of hypothalamic kiss1 levels by the suckling stimulus in the lactating rat. *J. Endocrinol.* 227 105–115. 10.1530/joe-15-0143 26446276

[B75] Hirano T. (1986). The spectrum of prolactin action in teleosts. *Prog. Clin. Biol. Res.* 205 53–74. 3513197

[B76] Hiroi J. McCormick S. D. (2012). New insights into gill ionocyte and ion transporter function in euryhaline and diadromous fish. *Respir. Physiol. Neurobiol.* 184 257–268. 10.1016/j.resp.2012.07.019 22850177

[B77] Holland L. Z. Albalat R. Azumi K. Benito-Gutierrez E. Blow M. J. Bronner-Fraser M. (2008). The amphioxus genome illuminates vertebrate origins and cephalochordate biology. *Genome Res.* 18 1100–1111. 18562680 10.1101/gr.073676.107 PMC2493399

[B78] Horseman N. D. Gregerson K. A. (2014). Prolactin actions. *J. Mol. Endocrinol.* 52 R95–R106. 24130130 10.1530/JME-13-0220

[B79] Ibarra F. Crambert S. Eklof A. C. Lundquist A. Hansell P. Holtback U. (2005). Prolactin, a natriuretic hormone, interacting with the renal dopamine system. *Kidney Int.* 68 1700–1707. 10.1111/j.1523-1755.2005.00586.x 16164646

[B80] Jorgacevski J. Stenovec M. Kreft M. Bajic A. Rituper B. Vardjan N. (2008). Hypotonicity and peptide discharge from a single vesicle. *Am. J. Physiol. Cell Physiol.* 295 C624–C631. 18632733 10.1152/ajpcell.00303.2008 PMC2544434

[B81] Kanematsu S. (1980). Crop sac stimulation after electrochemical stimulation of the brain in the pigeon. *Gen. Comp. Endocrinol.* 42 212–218. 10.1016/0016-6480(80)90190-2 6777239

[B82] Katayama Y. Sakamoto T. Saito K. Tsuchimochi H. Kaiya H. Watanabe T. (2018). Drinking by amphibious fish: convergent evolution of thirst mechanisms during vertebrate terrestrialization. *Sci. Rep.* 8:625. 10.1038/s41598-017-18611-4 PMC5766589 29330516

[B83] Keeler R. Wilson N. (1976). Vasopressin contamination as a cause of some apparent renal actions of prolactin. *Can. J. Physiol. Pharmacol.* 54 887–890. 10.1139/y76-124 1021223

[B84] Kennett J. E. Poletini M. O. Freeman M. E. (2008). Vasoactive intestinal polypeptide modulates the estradiol-induced prolactin surge by entraining oxytocin neuronal activity. *Brain Res.* 1196 65–73. 10.1016/j.brainres.2007.12.061 18234164 PMC2275054

[B85] Kline J. B. Clevenger C. V. (2001). Identification and characterization of the prolactin-binding protein in human serum and milk. *J. Biol. Chem.* 276 24760–24766. 10.1074/jbc.m011786200 11337493

[B86] Kokay I. C. Wyatt A. Phillipps H. R. Aoki M. Ectors F. Boehm U. (2018). Analysis of prolactin receptor expression in the murine brain using a novel prolactin receptor reporter mouse. *J. Neuroendocrinol.* 30:e12634. 10.1111/jne.12634 30040149

[B87] Kosonsiriluk S. Sartsoongnoen N. Chaiyachet O. A. Prakobsaeng N. Songserm T. Rozenboim I. (2008). Vasoactive intestinal peptide and its role in continuous and seasonal reproduction in birds. *Gen. Comp. Endocrinol.* 159 88–97. 10.1016/j.ygcen.2008.07.024 18761341

[B88] Kubokawa K. Tando Y. Roy S. (2010). Evolution of the reproductive endocrine system in chordates. *Integr. Comp. Biol.* 50 53–62. 10.1093/icb/icq047 21558187

[B89] Kuwayama T. Shimada K. Saito N. Ohkubo T. Sato K. Wada M. (1992). Effects of removal of chicks from hens on concentrations of prolactin, luteinizing hormone and oestradiol in plasma of brooding gifujidori hens. *J. Reprod. Fertil.* 95 617–622. 10.1530/jrf.0.0950617 1518016

[B90] Kwong A. K. Ng A. H. Leung L. Y. Man A. K. Woo N. Y. (2009). Effect of extracellular osmolality and ionic levels on pituitary prolactin release in euryhaline silver sea bream (*Sparus sarba*). *Gen. Comp. Endocrinol.* 160 67–75. 10.1016/j.ygcen.2008.10.024 19027016

[B91] Labella F. Dular R. Queen G. Vivian S. (1975). Anterior pituitary hormone release in vitro inversely related to extracellular osmolarity. *Endocrinology* 96 1559–1565. 10.1210/endo-96-6-1559 165067

[B92] Lam K. S. (1991). Vasoactive intestinal peptide in the hypothalamus and pituitary. *Neuroendocrinology* 53(Suppl. 1) 45–51. 10.1159/000125795 1901391

[B93] Le Tissier P. R. Hodson D. J. Martin A. O. Romano N. Mollard P. (2015). Plasticity of the prolactin (prl) axis: mechanisms underlying regulation of output in female mice. *Adv. Exp. Med. Biol.* 846 139–162. 10.1007/978-3-319-12114-7_6 25472537

[B94] Lea R. W. Sharp P. J. (1991). Effects of presence of squabs upon plasma concentrations of prolactin and lh and length of time of incubation in ringdoves on “extended” incubatory patterns. *Horm. Behav.* 25 275–282. 10.1016/0018-506x(91)90001-x 1937422

[B95] Ledoux J. E. Ruggiero D. A. Forest R. Stornetta R. Reis D. J. (1987). Topographic organization of convergent projections to the thalamus from the inferior colliculus and spinal cord in the rat. *J. Comp. Neurol.* 264 123–146. 10.1002/cne.902640110 2445791

[B96] Lee K. M. Kaneko T. Aida K. (2006). Prolactin and prolactin receptor expressions in a marine teleost, pufferfish takifugu rubripes. *Gen. Comp. Endocrinol.* 146 318–328. 10.1016/j.ygcen.2005.12.003 16430892

[B97] Leko A. H. Cservenak M. Dobolyi A. (2017a). Suckling induced insulin-like growth factor-1 (igf-1) release in mother rats. *Growth Horm. IGF Res.* 37 7–12. 10.1016/j.ghir.2017.10.003 29031906

[B98] Leko A. H. Cservenak M. Szabo E. R. Hanics J. Alpar A. Dobolyi A. (2017b). Insulin-like growth factor i and its binding protein-3 are regulators of lactation and maternal responsiveness. *Sci. Rep.* 7:3396. 10.1038/s41598-017-03645-5 PMC5469809 28611445

[B99] Li C. Chen P. Smith M. S. (1999). Neural populations in the rat forebrain and brainstem activated by the suckling stimulus as demonstrated by cfos expression. *Neuroscience* 94 117–129. 10.1016/s0306-4522(99)00236-5 10613502

[B100] Lignot J. H. Cutler C. P. Hazon N. Cramb G. (2002). Water transport and aquaporins in the european eel (*Anguilla anguilla*). *Symp. Soc. Exp. Biol.* 54 49–59. 14992144

[B101] Lin S. H. Miyata S. Matsunaga W. Kawarabayashi T. Nakashima T. Kiyohara T. (1998). Metabolic mapping of the brain in pregnant, parturient and lactating rats using fos immunohistochemistry. *Brain Res.* 787 226–236. 10.1016/s0006-8993(97)01484-4 9518626

[B102] Liu N. A. Liu Q. Wawrowsky K. Yang Z. Lin S. Melmed S. (2006). Prolactin receptor signaling mediates the osmotic response of embryonic zebrafish lactotrophs. *Mol. Endocrinol.* 20 871–880. 10.1210/me.2005-0403 16339273

[B103] Lonstein J. S. Stern J. M. (1997). Role of the midbrain periaqueductal gray in maternal nurturance and aggression: C-fos and electrolytic lesion studies in lactating rats. *J. Neurosci.* 17 3364–3378. 10.1523/jneurosci.17-09-03364.1997 9113892 PMC6573640

[B104] Lonstein J. S. Stern J. M. (1999). Effects of unilateral suckling on nursing behavior and c-fos activity in the caudal periaqueductal gray in rats. *Dev. Psychobiol.* 35 264–275. 10.1002/(sici)1098-2302(199912)35:4<264::aid-dev2>3.0.co;2-u 10573567

[B105] Lopez-Vicchi F. Ladyman S. R. Ornstein A. M. Gustafson P. Knowles P. Luque G. M. (2020). Chronic high prolactin levels impact on gene expression at discrete hypothalamic nuclei involved in food intake. *FASEB J.* 34 3902–3914. 10.1096/fj.201902357r 31944423

[B106] Lorenson M. Y. Jacobs L. S. (1987). Osmotic pressure regulation of prolactin and growth hormone release from bovine secretory granules. *Endocrinology* 120 365–372. 10.1210/endo-120-1-365 3780568

[B107] Loìpez M. A. C. Rodriìguez J. L. R. Garciìa M. R. (2003). “Physiological and pathological hyperprolactinemia: can we minimize errors in the clinical practice?,” in *Prolactin*, eds Nagy G. M. Toth B. E., (London: IntechOpen).

[B108] Lv C. Mo C. Liu H. Wu C. Li Z. Li J. (2018). Dopamine d2-like receptors (drd2 and drd4) in chickens: tissue distribution, functional analysis, and their involvement in dopamine inhibition of pituitary prolactin expression. *Gene* 651 33–43. 10.1016/j.gene.2018.01.087 29382572

[B109] Manzon L. A. (2002). The role of prolactin in fish osmoregulation: a review. *Gen. Comp. Endocrinol.* 125 291–310. 10.1006/gcen.2001.7746 11884075

[B110] March J. B. Sharp P. J. Wilson P. W. Sang H. M. (1994). Effect of active immunization against recombinant-derived chicken prolactin fusion protein on the onset of broodiness and photoinduced egg laying in bantam hens. *J. Reprod. Fertil.* 101 227–233. 10.1530/jrf.0.1010227 8064686

[B111] Massaro M. Setiawan A. N. Davis L. S. (2007). Effects of artificial eggs on prolactin secretion, steroid levels, brood patch development, incubation onset and clutch size in the yellow-eyed penguin (*Megadyptes antipodes*). *Gen. Comp. Endocrinol.* 151 220–229. 10.1016/j.ygcen.2007.01.034 17324416

[B112] Mayer-Gostan N. Hirano T. (1976). The effects of transecting the ixth and xth cranial nerves on hydromineral balance in the eel anguilla anguilla. *J. Exp. Biol.* 64 461–475. 932628 10.1242/jeb.64.2.461

[B113] McKinley M. J. Johnson A. K. (2004). The physiological regulation of thirst and fluid intake. *News Physiol. Sci.* 19 1–6. 10.1152/nips.01470.2003 14739394

[B114] Morrissey S. E. Newth T. Rees R. Barr A. Shora F. Laycock J. F. (2001). Renal effects of recombinant prolactin in anaesthetized rats. *Eur. J. Endocrinol.* 145 65–71. 10.1530/eje.0.1450065 11415854

[B115] Naef L. Woodside B. (2007). Prolactin/leptin interactions in the control of food intake in rats. *Endocrinology* 148 5977–5983. 10.1210/en.2007-0442 17872372

[B116] Nagaishi V. S. Cardinali L. I. Zampieri T. T. Furigo I. C. Metzger M. Donato J. Jr. (2014). Possible crosstalk between leptin and prolactin during pregnancy. *Neuroscience* 259 71–83. 10.1016/j.neuroscience.2013.11.050 24316468

[B117] Nagy G. M. Arendt A. Banky Z. Halasz B. (1992). Dehydration attenuates plasma prolactin response to suckling through a dopaminergic mechanism. *Endocrinology* 130 819–824. 10.1210/en.130.2.819 1733729

[B118] Nagy G. M. Bodnar I. Banky Z. Halasz B. (2005). Control of prolactin secretion by excitatory amino acids. *Endocrine* 28 303–308. 10.1385/endo:28:3:303 16388120

[B119] Namken S. Sinpru P. Kamkrathok B. Sartsoongnoen N. Chaiseha Y. (2017). Role of vasoactive intestinal peptide during the transition from incubation behavior to rearing behavior in the female native thai chicken. *Poult. Sci.* 96 3768–3774. 10.3382/ps/pex180 28938777

[B120] Neill J. D. Nagy G. M. (1994). “Prolactin secretion and its control,” in *Physiology of Reproduction*, eds Knobil E. Neill J. D., (New York: Raven Press), 1833–1860.

[B121] Nishimori K. Young L. J. Guo Q. Wang Z. Insel T. R. Matzuk M. M. (1996). Oxytocin is required for nursing but is not essential for parturition or reproductive behavior. *Proc. Natl. Acad. Sci. U.S.A.* 93 11699–11704. 10.1073/pnas.93.21.11699 8876199 PMC38121

[B122] Nobata S. Ando M. Takei Y. (2013). Hormonal control of drinking behavior in teleost fishes; insights from studies using eels. *Gen. Comp. Endocrinol.* 192 214–221. 10.1016/j.ygcen.2013.05.009 23707498

[B123] Ocampo Daza D. Larhammar D. (2018a). Evolution of the growth hormone, prolactin, prolactin 2 and somatolactin family. *Gen. Comp. Endocrinol.* 264 94–112. 10.1016/j.ygcen.2018.01.007 29339183

[B124] Ocampo Daza D. Larhammar D. (2018b). Evolution of the receptors for growth hormone, prolactin, erythropoietin and thrombopoietin in relation to the vertebrate tetraploidizations. *Gen. Comp. Endocrinol.* 257 143–160. 10.1016/j.ygcen.2017.06.021 28652136

[B125] Ohkubo T. (2017). Neuroendocrine control of broodiness. *Adv. Exp. Med. Biol.* 1001 151–171. 10.1007/978-981-10-3975-1_10 28980235

[B126] Olah S. Cservenak M. Keller D. Fazekas E. A. Renner E. Low P. (2018). Prolactin-induced and neuronal activation in the brain of mother mice. *Brain Struct. Funct.* 223 3229–3250. 10.1007/s00429-018-1686-1 29802523

[B127] On J. S. Chow B. K. Lee L. T. (2015). Evolution of parathyroid hormone receptor family and their ligands in vertebrate. *Front. Endocrinol. (Lausanne)* 6:28. 10.3389/fendo.2015.00028 25806022 PMC4354418

[B128] Ouhtit A. Morel G. Kelly P. A. (1993). Visualization of gene expression of short and long forms of prolactin receptor in the rat. *Endocrinology* 133 135–144. 10.1210/endo.133.1.8319561 8319561

[B129] Palkovits M. Usdin T. B. Makara G. B. Dobolyi A. (2010). Tuberoinfundibular peptide of 39 residues- immunoreactive fibers in the zona incerta and the supraoptic decussations terminate in the neuroendocrine hypothalamus. *Neurochem. Res.* 35 2078–2085. 10.1007/s11064-010-0292-2 20972828 PMC3388614

[B130] Parker S. L. Armstrong W. E. Sladek C. D. Grosvenor C. E. Crowley W. R. (1991). Prolactin stimulates the release of oxytocin in lactating rats: evidence for a physiological role via an action at the neural lobe. *Neuroendocrinology* 53 503–510. 10.1159/000125764 1883415

[B131] Peaker M. Phillips J. G. Wright A. (1970). The effect of prolactin on the secretory activity of the nasal salt-gland of the domestic duck (*Anas platyrhynchos*). *J. Endocrinol.* 47 123–127. 10.1677/joe.0.0470123 5428908

[B132] Phillipps H. R. Yip S. H. Grattan D. R. (2019). Patterns of prolactin secretion. *Mol. Cell Endocrinol.* 502:110679. 10.1016/j.mce.2019.110679 31843563

[B133] Pi X. Voogt J. L. (2000). Effect of suckling on prolactin receptor immunoreactivity in the hypothalamus of the rat. *Neuroendocrinology* 71 308–317. 10.1159/000054551 10859493

[B134] Pi X. J. Grattan D. R. (1999). Increased prolactin receptor immunoreactivity in the hypothalamus of lactating rats. *J. Neuroendocrinol.* 11 693–705. 10.1046/j.1365-2826.1999.00386.x 10447808

[B135] Pickford G. E. Phillips J. G. (1959). Prolactin, a factor in promoting survival of hypophysectomized killifish in fresh water. *Science* 130 454–455. 10.1126/science.130.3373.454 13675773

[B136] Proudman J. A. Opel H. (1988). Stimulation of prolactin secretion from turkey anterior pituitary cells in culture. *Proc. Soc. Exp. Biol. Med.* 187 448–454. 10.3181/00379727-187-42687 3353393

[B137] Radhakrishnan A. Raju R. Tuladhar N. Subbannayya T. Thomas J. K. Goel R. (2012). A pathway map of prolactin signaling. *J. Cell Commun. Signal.* 6 169–173. 22684822 10.1007/s12079-012-0168-0 PMC3421022

[B138] Reynolds J. D. Goodwin N. B. Freckleton R. P. (2002). Evolutionary transitions in parental care and live bearing in vertebrates. *Philos. Trans. R Soc. Lond. B Biol. Sci.* 357 269–281. 10.1098/rstb.2001.0930 11958696 PMC1692951

[B139] Richard-Yris M. A. Sharp P. J. Wauters A. M. Guemene D. Richard J. P. Foraste M. (1998). Influence of stimuli from chicks on behavior and concentrations of plasma prolactin and luteinizing hormone in incubating hens. *Horm. Behav.* 33 139–148. 10.1006/hbeh.1998.1444 9647939

[B140] Romano N. Yip S. H. Hodson D. J. Guillou A. Parnaudeau S. Kirk S. (2013). Plasticity of hypothalamic dopamine neurons during lactation results in dissociation of electrical activity and release. *J. Neurosci.* 33 4424–4433. 10.1523/jneurosci.4415-12.2013 23467359 PMC6704969

[B141] Romanov M. N. Talbot R. T. Wilson P. W. Sharp P. J. (2002). Genetic control of incubation behavior in the domestic hen. *Poult. Sci.* 81 928–931. 10.1093/ps/81.7.928 12162351

[B142] Rosenblatt J. S. (2003). Outline of the evolution of behavioral and nonbehavioral patterns of parental care among the vertebrates: critical characteristics of mammalian and avian parental behavior. *Scand. J. Psychol.* 44 265–271. 10.1111/1467-9450.00344 12914590

[B143] Royle N. J. Russell A. F. Wilson A. J. (2014). The evolution of flexible parenting. *Science* 345 776–781. 10.1126/science.1253294 25124432

[B144] Rozenboim I. Tabibzadeh C. Silsby J. L. el Halawani M. E. (1993). Effect of ovine prolactin administration on hypothalamic vasoactive intestinal peptide (vip), gonadotropin releasing hormone i and ii content, and anterior pituitary vip receptors in laying turkey hens. *Biol. Reprod.* 48 1246–1250. 10.1095/biolreprod48.6.1246 8391330

[B145] Sage M. Bern H. A. (1971). Cytophysiology of the teleost pituitary. *Int. Rev. Cytol.* 31 339–376. 10.1016/s0074-7696(08)60062-5 4333817

[B146] Saiardi A. Bozzi Y. Baik J. H. Borrelli E. (1997). Antiproliferative role of dopamine: loss of d2 receptors causes hormonal dysfunction and pituitary hyperplasia. *Neuron* 19 115–126. 10.1016/s0896-6273(00)80352-9 9247268

[B147] Sakamoto T. McCormick S. D. (2006). Prolactin and growth hormone in fish osmoregulation. *Gen. Comp. Endocrinol.* 147 24–30. 10.1016/j.ygcen.2005.10.008 16406056

[B148] Salais-Lopez H. Lanuza E. Agustin-Pavon C. Martinez-Garcia F. (2017). Tuning the brain for motherhood: prolactin-like central signalling in virgin, pregnant, and lactating female mice. *Brain Struct. Funct.* 222 895–921. 10.1007/s00429-016-1254-5 27344140

[B149] Sandra O. Le Rouzic P. Cauty C. Edery M. Prunet P. (2000). Expression of the prolactin receptor (tiprl-r) gene in tilapia oreochromis niloticus: tissue distribution and cellular localization in osmoregulatory organs. *J. Mol. Endocrinol.* 24 215–224. 10.1677/jme.0.0240215 10750022

[B150] Santos C. R. Ingleton P. M. Cavaco J. E. Kelly P. A. Edery M. Power D. M. (2001). Cloning, characterization, and tissue distribution of prolactin receptor in the sea bream (*Sparus aurata*). *Gen. Comp. Endocrinol.* 121 32–47. 10.1006/gcen.2000.7553 11161768

[B151] Sapsford T. J. Kokay I. C. Ostberg L. Bridges R. S. Grattan D. R. (2012). Differential sensitivity of specific neuronal populations of the rat hypothalamus to prolactin action. *J. Comp. Neurol.* 520 1062–1077. 10.1002/cne.22775 21953590 PMC3936877

[B152] Sartsoongnoen N. Kosonsiriluk S. Prakobsaeng N. Songserm T. Rozenboim I. Halawani M. E. (2008). The dopaminergic system in the brain of the native thai chicken, gallus domesticus: localization and differential expression across the reproductive cycle. *Gen. Comp. Endocrinol.* 159 107–115. 10.1016/j.ygcen.2008.08.002 18765240

[B153] Sauve D. Woodside B. (1996). The effect of central administration of prolactin on food intake in virgin female rats is dose-dependent, occurs in the absence of ovarian hormones and the latency to onset varies with feeding regimen. *Brain Res.* 729 75–81. 10.1016/s0006-8993(96)00227-2 8874878

[B154] Scott N. Prigge M. Yizhar O. Kimchi T. (2015). A sexually dimorphic hypothalamic circuit controls maternal care and oxytocin secretion. *Nature* 525 519–522. 10.1038/nature15378 26375004

[B155] Seale A. P. Watanabe S. Grau E. G. (2012). Osmoreception: perspectives on signal transduction and environmental modulation. *Gen. Comp. Endocrinol.* 176 354–360. 10.1016/j.ygcen.2011.10.005 22036842

[B156] Shu Y. Lou Q. Dai Z. Dai X. He J. Hu W. (2016). The basal function of teleost prolactin as a key regulator on ion uptake identified with zebrafish knockout models. *Sci. Rep.* 6:18597. 10.1038/srep18597 PMC4698586 26726070

[B157] Sinpru P. Sartsoongnoen N. Rozenboim I. Porter T. E. El Halawani M. E. Chaiseha Y. (2018). The effects of replacing eggs with chicks on mesotocin, dopamine, and prolactin in the native thai hen. *Gen. Comp. Endocrinol.* 263 32–42. 10.1016/j.ygcen.2018.04.013 29660308

[B158] Sjoeholm A. Bridges R. S. Grattan D. R. Anderson G. M. (2011). Region-, neuron-, and signaling pathway-specific increases in prolactin responsiveness in reproductively experienced female rats. *Endocrinology* 152 1979–1988. 10.1210/en.2010-1220 21363933 PMC3075931

[B159] Smiley K. O. (2019). Prolactin and avian parental care: new insights and unanswered questions. *Horm. Behav.* 111 114–130. 10.1016/j.yhbeh.2019.02.012 30802443

[B160] Smiley K. O. Adkins-Regan E. (2016). Relationship between prolactin, reproductive experience, and parental care in a biparental songbird, the zebra finch (*Taeniopygia guttata*). *Gen. Comp. Endocrinol.* 232 17–24. 10.1016/j.ygcen.2015.11.012 26602378

[B161] Soares M. J. Konno T. Alam S. M. (2007). The prolactin family: effectors of pregnancy-dependent adaptations. *Trends Endocrinol. Metab.* 18 114–121. 10.1016/j.tem.2007.02.005 17324580

[B162] Specker J. L. Kishida M. Huang L. King D. S. Nagahama Y. Ueda H. (1993). Immunocytochemical and immunogold localization of two prolactin isoforms in the same pituitary cells and in the same granules in the tilapia (*Oreochromis mossambicus*). *Gen. Comp. Endocrinol.* 89 28–38. 10.1006/gcen.1993.1006 7679085

[B163] Suarez-Bregua P. Cal L. Canestro C. Rotllant J. (2017). PTH reloaded: a new evolutionary perspective. *Front. Physiol.* 8:776. 10.3389/fphys.2017.00776 29062283 PMC5640766

[B164] Suzuki K. Koizumi N. Hirose H. Hokao R. Takemura N. Motoyoshi S. (2000). Changes in plasma arginine vasopressin concentration during lactation in rats. *Comp Med* 50 277–280. 10894491

[B165] Szawka R. E. Ribeiro A. B. Leite C. M. Helena C. V. Franci C. R. Anderson G. M. (2011). Kisspeptin regulates prolactin release through hypothalamic dopaminergic neurons. *Endocrinology* 151 3247–3257. 10.1210/en.2009-1414 20410200

[B166] Tacon P. Baroiller J. F. Le Bail P. Y. Prunet P. Jalabert B. (2000). Effect of egg deprivation on sex steroids, gonadotropin, prolactin, and growth hormone profiles during the reproductive cycle of the mouthbrooding cichlid fish oreochromis niloticus. *Gen. Comp. Endocrinol.* 117 54–65. 10.1006/gcen.1999.7388 10620423

[B167] Takei Y. Hiroi J. Takahashi H. Sakamoto T. (2014). Diverse mechanisms for body fluid regulation in teleost fishes. *Am. J. Physiol. Regul. Integr. Comp. Physiol.* 307 R778–R792. 24965789 10.1152/ajpregu.00104.2014

[B168] Tindal J. S. Knaggs G. S. (1969). An ascending pathway for release of prolactin in the brain of the rabbit. *J. Endocrinol.* 45 111–120. 10.1677/joe.0.0450111 5388353

[B169] Tindal J. S. Knaggs G. S. (1972). Pathways in the forebrain of the rabbit concerned with the release of prolactin. *J. Endocrinol.* 52 253–262. 10.1677/joe.0.0520253 5015381

[B170] Tindal J. S. Knaggs G. S. (1977). Pathways in the forebrain of the rat concerned with the release of prolactin. *Brain Res.* 119 211–221. 10.1016/0006-8993(77)90101-9 401464

[B171] Tong Z. Pitts G. R. You S. Foster D. N. El Halawani M. E. (1998). Vasoactive intestinal peptide stimulates turkey prolactin gene expression by increasing transcription rate and enhancing mrna stability. *J. Mol. Endocrinol.* 21 259–266. 10.1677/jme.0.0210259 9845667

[B172] Usdin T. B. Dobolyi A. Ueda H. Palkovits M. (2003). Emerging functions for tuberoinfundibular peptide of 39 residues. *Trends Endocrinol. Metab.* 14 14–19. 10.1016/s1043-2760(02)00002-4 12475607

[B173] Van Goor F. Zivadinovic D. Martinez-Fuentes A. J. Stojilkovic S. S. (2001). Dependence of pituitary hormone secretion on the pattern of spontaneous voltage-gated calcium influx. Cell type-specific action potential secretion coupling. *J. Biol. Chem.* 276 33840–33846. 10.1074/jbc.m105386200 11457854

[B174] Voogt J. L. Lee Y. Yang S. Arbogast L. (2001). Regulation of prolactin secretion during pregnancy and lactation. *Prog. Brain Res.* 133 173–185. 10.1016/s0079-6123(01)33013-3 11589129

[B175] Walker C. D. Toufexis D. J. Burlet A. (2001). Hypothalamic and limbic expression of crf and vasopressin during lactation: implications for the control of acth secretion and stress hyporesponsiveness. *Prog. Brain Res.* 133 99–110. 10.1016/s0079-6123(01)33008-x 11589148

[B176] Wan X. P. Xie P. Bu Z. Zou X. T. Gong D. Q. (2019). Prolactin induces lipid synthesis of organ-cultured pigeon crops. *Poult. Sci.* 98 1842–1853. 10.3382/ps/pey540 30590797

[B177] Watanabe H. Davis J. B. Smart D. Jerman J. C. Smith G. D. Hayes P. (2002). Activation of trpv4 channels (hvrl-2/mtrp12) by phorbol derivatives. *J. Biol. Chem.* 277 13569–13577. 10.1074/jbc.m200062200 11827975

[B178] Watanabe S. Hirano T. Grau E. G. Kaneko T. (2009). Osmosensitivity of prolactin cells is enhanced by the water channel aquaporin-3 in a euryhaline mozambique tilapia (*Oreochromis mossambicus*). *Am. J. Physiol. Regul. Integr. Comp. Physiol.* 296 R446–R453. 19109372 10.1152/ajpregu.90435.2008

[B179] Watanabe S. Kaneko T. Aida K. (2005). Aquaporin-3 expressed in the basolateral membrane of gill chloride cells in mozambique tilapia *Oreochromis mossambicus* adapted to freshwater and seawater. *J. Exp. Biol.* 208 2673–2682. 10.1242/jeb.01684 16000537

[B180] Weber G. M. Seale A. P. Richman I. N. Stetson M. H. Hirano T. Grau E. G. (2004). Hormone release is tied to changes in cell size in the osmoreceptive prolactin cell of a euryhaline teleost fish, the tilapia, *Oreochromis mossambicus*. *Gen. Comp. Endocrinol.* 138 8–13. 10.1016/j.ygcen.2004.04.006 15242746

[B181] Whittington C. M. Wilson A. B. (2013). The role of prolactin in fish reproduction. *Gen. Comp. Endocrinol.* 191 123–136. 10.1016/j.ygcen.2013.05.027 23791758

[B182] Witcher J. A. Freeman M. E. (1985). The proestrous surge of prolactin enhances sexual receptivity in the rat. *Biol. Reprod.* 32 834–839. 10.1095/biolreprod32.4.834 4039953

[B183] Wu Z. Autry A. E. Bergan J. F. Watabe-Uchida M. Dulac C. G. (2014). Galanin neurons in the medial preoptic area govern parental behaviour. *Nature* 509 325–330. 10.1038/nature13307 24828191 PMC4105201

[B184] Xu H. Shen X. Zhou M. Fang M. Zeng H. Nie Q. (2010). The genetic effects of the dopamine d1 receptor gene on chicken egg production and broodiness traits. *BMC Genet.* 11:17. 10.1186/1471-2156-11-17 20199684 PMC2848132

[B185] Yamaguchi Y. Moriyama S. Lerner D. T. Grau E. G. Seale A. P. (2016). Autocrine positive feedback regulation of prolactin release from tilapia prolactin cells and its modulation by extracellular osmolality. *Endocrinology* 157 3505–3516. 10.1210/en.2015-1969 27379370 PMC6285229

[B186] Yip S. H. Romano N. Gustafson P. Hodson D. J. Williams E. J. Kokay I. C. (2019). Elevated prolactin during pregnancy drives a phenotypic switch in mouse hypothalamic dopaminergic neurons. *Cell Rep.* 26 1787–1799. 30759390 10.1016/j.celrep.2019.01.067

[B187] Youngren O. M. el Halawani M. E. Phillips R. E. Silsby J. L. (1989). Effects of preoptic and hypothalamic lesions in female turkeys during a photoinduced reproductive cycle. *Biol. Reprod.* 41 610–617. 10.1095/biolreprod41.4.610 2620072

[B188] Youngren O. M. el Halawani M. E. Silsby J. L. Phillips R. E. (1991). Intracranial prolactin perfusion induces incubation behavior in turkey hens. *Biol. Reprod.* 44 425–431. 10.1095/biolreprod44.3.425 2015361

